# Cytoskeletal Protein Palladin in Adult Gliomas Predicts Disease Incidence, Progression, and Prognosis

**DOI:** 10.3390/cancers14205130

**Published:** 2022-10-19

**Authors:** Ori Mayer, Joshua Bugis, Daria Kozlova, Aviv Leemann, Shahar Mansur, Ilan Peerutin, Noga Mendelovich, Meital Mazin, Dinorah Friedmann-Morvinski, Noam Shomron

**Affiliations:** 1 Department of Cell and Developmental Biology, Faculty of Medicine, Tel-Aviv University, Tel-Aviv 6997801, Israel; 2 Department of Pathology, Rabin Medical Center, Beilinson Campus, Petah Tikva 4941492, Israel; 3 The School of Neurobiology, Biochemistry and Biophysics, George S. Wise Faculty of Life Sciences, Tel Aviv University, Tel-Aviv 6997801, Israel; 4 Sagol School of Neuroscience, Tel-Aviv University, Tel-Aviv 6997801, Israel; 5 Edmond J. Safra Center for Bioinformatics, Tel Aviv University, Tel-Aviv 6997801, Israel

**Keywords:** PALLD, central nervous system neoplasm, diagnostic marker, prognostic marker, glioblastoma

## Abstract

**Simple Summary:**

Glioma is a tumor originating from cells supporting the brain and represents a major health challenge. Palladin is a structural protein widely expressed in mammalian tissues and has a pivotal role in cytoskeletal dynamics in health and disease. Palladin is linked to the progression of breast, pancreatic, and renal cancers. However, the role of palladin in gliomas is yet unknown. In this work, we aimed to shed light on palladin’s role in glioma tumors using publicly available data, along with samples obtained from humans and mice. Our findings indicate that palladin expression might be linked to adult glioma progression and is associated with a worse prognosis. Overall, our results introduce the possibility of using palladin as a diagnostic and prognostic marker, as well as a potential future therapeutic target.

**Abstract:**

Brain tumors comprise over 100 types of masses, differing in the following: location; patient age; molecular, histological, and immunohistochemical characteristics; and prognosis and treatment. Glioma tumors originate from neuroglia, cells supporting the brain. Palladin, a structural protein widely expressed in mammalian tissues, has a pivotal role in cytoskeletal dynamics and motility in health and disease. Palladin is linked to the progression of breast, pancreatic, and renal cancers. In the central nervous system, palladin is involved in embryonic development, neuronal maturation, the cell cycle, differentiation, and apoptosis. However, the role of palladin in brain tumors is unknown. In this work, we explored palladin’s role in glioma. We analyzed clinical data, along with bulk and single-cell gene expression. We then validated our results using IHC staining of tumor samples, together with qRT-PCR of glioma cell lines. We determined that wild-type palladin-4 is overexpressed in adult gliomas and is correlated with a decrease in survival. Palladin expression outperformed clinically used prognostic markers and was most prominent in glioblastoma. Finally, we showed that palladin originates from the malignant cell population. Our findings indicate that palladin expression might be linked to adult glioma progression and is associated with prognosis.

## 1. Introduction

Gliomas, which originate from the brain’s support cells, or neuroglia, comprise 23–25% of all brain tumors, and 80% of malignant brain tumors [1]. The World Health Organization (WHO) classifies glioma tumors according to the following: their molecular, histological, and immunohistochemical characteristics; whether the tumor is diffuse or circumscribed; and its occurrence in adult or pediatric patients. To diagnose central nervous system (CNS) tumors, a pathologist determines the histology of the tumor, grades it from 1–4, and reports on the molecular characteristics of interest. Finally, considering all the features of the lesion, an integrated diagnosis is assigned following the WHO CNS5 guidelines [2]. Typical survival time is in the range of 1–10 years. For glioblastomas, the most common form of glioma, the 5-year survival time is only 6.8% [1].

The current leading prognostic factors of glioma are age, Karnofsky performance score (KPS), and tumor grade. The number of glioma lesions and the degree of surgical resection also impact prognosis [3,4]. Several genetic alterations serve as prognostic factors for glioma. Loss of heterozygosity of 1p/19q is considered a favorable prognostic factor, though this association is stronger in oligodendrogliomas than in astrocytomas and glioblastomas, and is, therefore, also used in diagnosis [5,6,7]. Gain of function mutations in the TP53 gene, causing overexpression of p53 protein, is an adverse prognostic factor associated with shorter overall survival [8]. Isocitrate dehydrogenase 1 (IDH1) and IDH2 mutations are favorable prognostic factors and can facilitate diagnosing astrocytomas and oligodendrogliomas, whereas IDH-wildtype is associated with lower overall survival and characterizes glioblastomas [9]. Promoter methylation of O6-methylguanine DNA methyltransferase (MGMT) is a favorable prognostic factor that is associated with enhanced sensitivity to alkylating agents in chemotherapy [10,11]. Lastly, ATRX mutations, which occur most often in astrocytoma, are associated with wildtype 1p/19q and with mutations in IDH1/2 and TP53, and may be involved in alternative lengthening of telomeres, and, thus, contributing to genomic instability [12,13].

The current standard of care for gliomas is surgical resection, followed by chemotherapy with temozolomide (TMZ) and radiation therapy [14]. Tumor treating fields (TTFields) are alternating electric fields that stunt tumor growth by interfering with the cell cycle [15]. Clinical trials have shown that TTFields, in combination with TMZ, improve the overall median survival of patients with glioblastoma by 5 months compared to TMZ alone [16]. The drug bevacizumab employs a humanized antibody that targets human vascular endothelial growth factor, resulting in decreased tumor vascularization and, consequently, reduced tumor proliferation [14,17]. Despite advances in treatments, high-grade gliomas (HGG) remain largely incurable.

Palladin, encoded in the PALLD gene, is a widely expressed protein in mammalian tissues. This protein has pivotal roles in cytoskeletal organization and motility in developing, normal, and diseased tissues [18,19]. Palladin is localized to both highly motile and actin rich structures, such as stress fibers, focal adhesions, dorsal ruffles, podosomes, Z-discs, invadopodia, and filopodia [18,20]. Palladin acts as a major scaffolding protein, by recruiting other actin-related proteins, such as profilin, VASP, α-actinin, ezrin, PDLIM1, Eps8, and LASP-1 [21,22,23,24,25,26,27]. Palladin also promotes actin filament nucleation and crosslinking, thus supporting stronger fibers in higher numbers. Taken together, palladin is a major player in cytoskeletal dynamics [18,28,29,30,31]. It has been linked to the progression and aggressive behavior of breast [32], pancreatic [33], ovarian [34], colorectal [35], and renal cancers [36]. Previously, in an orthotopic breast cancer metastasis mice model, we showed that local delivery of miR-96/miR-182, bound to gold nanoparticles and embedded in a hydrogel, reduced metastasis by downregulating palladin expression [37]. In the CNS, palladin is expressed in the neural plate, neural progenitor cells, cortical neurons, and astrocytes [38,39,40]. The protein is involved in embryonic development, neuronal maturation, the cell cycle, differentiation, and apoptosis [38,41,42,43]. The role of palladin in brain tumors is currently unknown. In this study, we investigated the link between palladin expression and glioma progression, and we assessed palladin’s potential as a marker of disease aggressiveness.

## 2. Materials and Methods

### 2.1. Genomic Mutation, Bulk Gene Expression, Clinical Data, and Survival Analysis

The genomic, bulk gene expression, clinical data, and the overall survival data of tumor and healthy samples used in this study were based on data generated from several datasets. The primary datasets were The Cancer Genome Atlas Pan-Cancer (TCGA-PANCAN), TCGA Glioblastoma Multiforme and Lower Grade Glioma (TCGA-GBMLGG) [44], and the Genotype-Tissue Expression (GTEx) [45] datasets (Supplementary Table S1 contains a comprehensive list of the datasets used). The data were accessed by the UCSC Xena browser and GlioVis server (accessed on 13 July 2020 and 15 February 2022, respectively) [46,47]. Analysis and visualization were performed using either BioRender (https://biorender.com/ (accessed on 22 June 2022)) or GraphPad Prism 9.3.1 (Graphpad Software, San Diego, CA, USA). Multivariate Cox regression analysis was done via in-house scripts using R version 4.1.1 (R Foundation, Vienna, Austria).

### 2.2. Cell Culture

Murine glioblastoma stem cells 005 and 007 were grown in Dulbecco’s Modified Eagle’s Medium (DMEM)/F12 media (Gibco, Waltham, MA, USA), supplemented with GlutaMAX 1:100 (Gibco), 100 units/mL penicillin, 50 mg/mL streptomycin, N2 supplement 1:100 (Gibco), 2.5 µg/mL heparin (Sigma, St. Louis, MO, USA), 20 ng/mL fibroblast growth factor (Peprotech, Cranbury, NJ, USA), and 20 ng/mL epidermal growth factor (Peprotech). AFFR53 and AGR53 murine glioblastoma cells were grown in DMEM, high glucose (Biological Industries, Mateh Asher, Israel) supplemented with 10% fetal bovine serum (Biological Industries), 2 mM sodium pyruvate (Biological Industries), 100 units/mL penicillin, and 50 mg/mL streptomycin. Cells were incubated at 37 °C in a 5% CO_2_ atmosphere. Before use, each cell line was confirmed to have no mycoplasma contamination using the EZ-PCR mycoplasma test kit (Biological Industries) [35].

### 2.3. RNA Extraction and Quantitative Reverse Transcription-Polymerase Chain Reaction (qRT-PCR)

Total RNA from cell lines was extracted using TRIzol reagent, according to the manufacturer’s instructions (Invitrogen, Thermo Fisher Scientific, Waltham, MA, USA). Reverse transcription reaction was conducted using High-Capacity cDNA Reverse Transcription Kit with random primers (ABI). The expression of mRNA was tested using SYBR Green PCR Master Mix (ABI). PCR amplification and reading was done in triplicates using the StepOnePlus thermal cycler (ABI). Palladin expression values were calculated based on the comparative threshold cycle (Ct) method and normalized to glyceraldehyde 3-phosphate dehydrogenase (GAPDH).

### 2.4. Animal Studies and Fluorescent Confocal Microscopy of Mouse Glioblastoma Tumors

C57BL/6J mice were purchased from Envigo, Jerusalem, Israel. All experiments involving animals were approved by the Tel Aviv University Institutional Animal Care and Use Committee. All the mice used in this study were 8–16 weeks old when tumors were induced and bred under pathogen-free conditions. All animals were housed in individually ventilated cages (5 mice per cage) with autoclaved ASPEN wood chips bedding and provided with food and drinking water ad libitum with 12/12-h light/dark cycle. A total of 3 × 10^5^ 005 cells stably expressing enhanced green fluorescent protein (GFP) were stereotaxically injected into the hippocampus of the mice. After allowing the tumors to develop for 27–35 days, the mice were perfused with 1× PBS and fixed with 4% paraformaldehyde. Brains were collected and coronal sections (30–40 μm) were cut using a HM450 Microtome (Thermo Fisher Scientific). Floating sections were blocked for 2 h using a goat anti-mouse-HRP antibody (Jackson ImmnoResearch, Chester County, PA, USA, Cat No. 115-035-166, Dilution 1:100) and then incubated overnight at 4 °C with a mouse anti-palladin monoclonal antibody (Novus, Cat No. NBP1-25959, Dilution: 1:100), followed by goat anti-mouse-AlexaFluor647 (Abcam, Cat No. ab150115, dilution 1:100). Nuclei were counterstained with DAPI (Molecular Probes, Eugene, OR, USA) at 1 μg/mL. Images were acquired using a Leica TCS SP8 confocal microscope (Leica Microsystems, Wetzlar, Germany). This comprised a 405 nm diode laser focused through an HC PL APO 63× and a 1.4-numerical aperture oil immersion objective. Excitation and emission ranges were: DAPI (412–450), GFP (495–550), and AlexaFluor647 (647–665). Images were analyzed using Fiji/ImageJ 1.53o.

### 2.5. Immunohistochemical Staining of Palladin in Human Tissue Microarrays

Formalin-fixed paraffin-embedded human tissue microarrays (TMA) with CNS pathologies were obtained from US Biomax, Inc. (GL2081, Derwood, MD, USA) and stained using BOND-III (Leica Biosystems). Anti-palladin primary antibody was diluted 1:100 and incubated for 1.5 h. Post primary antibodies and polymer were both added and incubated for 1 h one at a time. Last, 3,3′-diaminobenzidine (DAB) was added followed by hematoxylin counterstain. The slide was analyzed by a pathologist. All the incubation steps were carried out at room temperature using reagents in standard supplied with BOND-III.

### 2.6. Single-Cell Gene Expression Data

Single-cell gene expression data of astrocytoma [48] and glioblastoma multiforme (GBM) [49] studies were accessed via the Single Cell Portal (https://singlecell.broadinstitute.org/single_cell (accessed on 14 December 2021)). For the purpose of finding genes similar to palladin, we used GeneCardsSuite’s GeneLikeMe server (https://glm.genecards.org/about (accessed on 3 May 2022)). Analysis and visualization were done via in-house scripts using R version 4.1.1 packages and written in RStudio version 1.4.1717.

### 2.7. In-Silico Flow Cytometry of Astrocytoma Tumors

To impute cell fractions in IDH1-mutated astrocytoma tumors, we used the CIBERSORTx server (https://cibersortx.stanford.edu/ (accessed on 18 August 2021)). Single cell RNA-seq of IDH-mutant astrocytoma data was downloaded from the Single Cell Portal and used to create a signature matrix. This signature matrix was then employed to impute cell fractions in samples from IDH1-mutated astrocytoma tumors from the TCGA-LGG dataset. Finally, we analyzed differences in cell fractions between tumors of different histological grades.

### 2.8. Enrichment Analysis of Gene Co-Expression with Palladin

Co-expression data were accessed and downloaded from the SEEK [Human] server (https://seek.princeton.edu/seek/ (accessed on 8 August 2021)). We conducted two distinct queries with PALLD as the query gene, one limiting the search space to astrocytoma-related datasets and the other limiting to GBM-related datasets. We then filtered out genes with a |co-expression Z score| ≥ 1 and *p* < 0.01. Analysis and visualization were done via in-house scripts using R version 4.1.1 packages and written in RStudio version 1.4.1717.

### 2.9. Statistical Analyses

Data are presented as violin plots or bar plots with median or mean ± SEM (standard error of the mean), respectively, as was calculated by GraphPad Prism 9.3.1, which was also used for visualization. Statistical Analyses of gene expression and clinical data included initial outlier identification and removal using the ROUT (Q = 1%) method followed by the Shapiro–Wilk normality test. Unpaired t and Mann–Whitney tests were used to compare between groups. Ordinary one-way ANOVA and Kruskal–Wallis tests were used in analyses of more than two groups, together with Holm–Šídák and Dunn’s multiple comparison test, respectively. In all the tests, an α = 0.05 was used.

## 3. Results

### 3.1. Palladin Is Overexpressed in Adult Glioma Tumors and Is Correlated with Shorter Overall Survival

**Figure 1:**
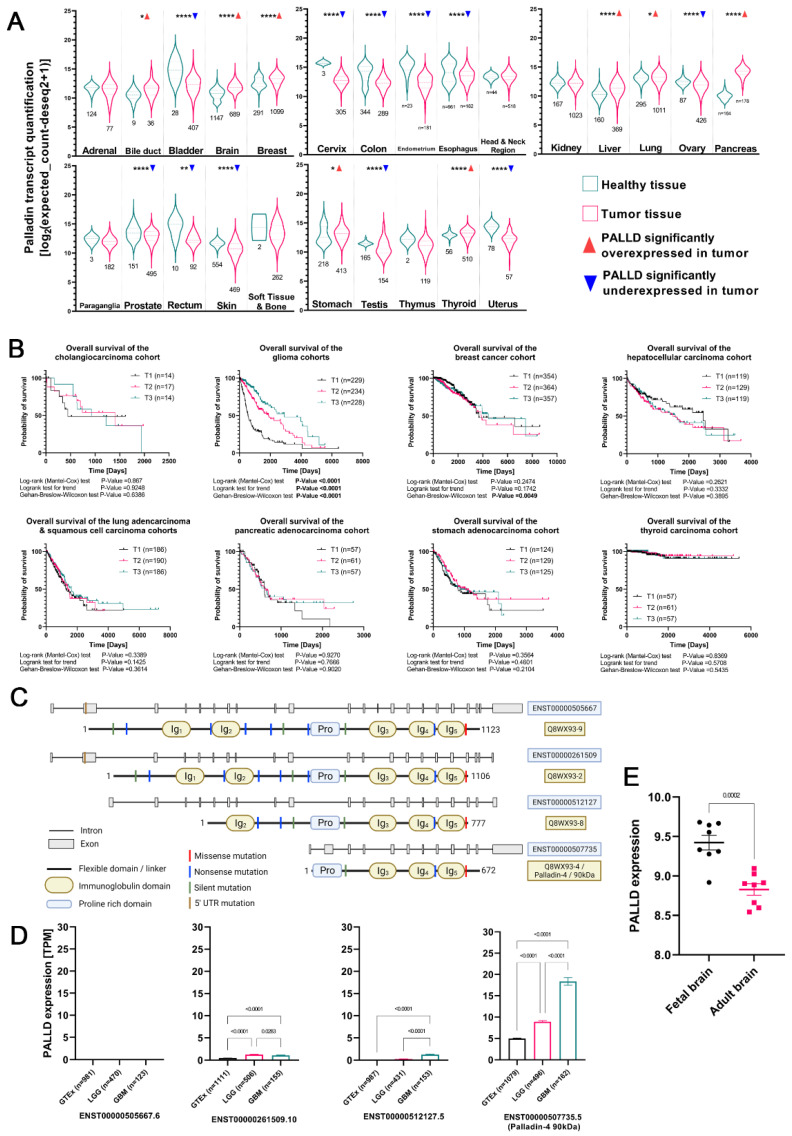
Wild-type palladin-4 mRNA is overexpressed in adult gliomas and is correlated with decreased survival. (**A**) Palladin expression in healthy and tumor samples. (**B**) Overall survival of all palladin-overexpressing tumors in (**A**), stratified into three groups based on expression level. (**C**) Schematic representation of palladin’s complete coding transcripts, their protein products, and somatic mutations. (**D**) Expression levels of the four coding palladin isoforms. (**E**) Comparison of palladin expression in fetal brains and adult brains. The data are shown as (**A**) the median with the first and third quartiles (**D**,**E**) means ± SEM. * = *p* < 0.05, ** = *p* < 0.01, **** = *p* < 0.0001, (**A**) unpaired *t*-test or Mann–Whitney U test, depending on the Shapiro–Wilk normality test result, (**B**) log–rank test, log–rank test for trend, and Gehan–Breslow–Wilcoxon test, (**D**) unpaired t-test and Kruskal–Wallis test, (**E**) unpaired t-test.

To identify cancers in which palladin may be involved in their pathogenesis, we analyzed the transcription of the PALLD gene in tumor and healthy tissues. Expression data from 25 organs were acquired from the TCGA (tumor samples) and GTEx (healthy tissue samples) datasets; the sample sizes ranged from two to 1098 per organ after outlier removal. We identified eight tumor types with significant PALLD overexpression relative to healthy tissue: bile duct, brain, breast, liver, lung, pancreas, stomach, and thyroid (Figure 1A and Table 1). Tumors in eleven organs exhibited significant downregulation: the bladder, cervix, colon, endometrium, esophagus, ovary, prostate, rectum, skin, testis, and uterus. Tumors from the remaining body regions showed no significant difference in expression between cancer and normal tissue.

Previous work done in our lab identified palladin as having a tumor promoting function; therefore, we focused on palladin-overexpressing tumors. We analyzed overall survival data in palladin-overexpressing tumors. Survival data from each of the eight palladin-overexpressing TCGA cohorts were stratified into three tertiles, based on palladin expression levels, and analyzed. Significant differences in survival probability between high, moderate, and low palladin-expressing groups were only observed in brain tumors (the combined glioma cohort comprised by the low-grade glioma (LGG) and GBM sub-cohorts) (χ^2^_Mental-Cox_ = 115.7, df = 2, *p* < 0.0001, Figure 1B). The difference in survival mimics a dose response relation; median survival was 548, 1886, and 2907 days in tumors with high, medium, and low levels of palladin, respectively. To further validate our results, we analyzed patient survival curves with respect to palladin expression, of all the adult glioma datasets available on the GlioVis website, after determining the optimal cutoff. Of the 17 datasets that included both survival and palladin expression data, 10 exhibited a significant decrease in survival in palladin-overexpressing samples and one showed a similar non-significant trend. The six remaining datasets exhibited four significant and two non-significant trends of increased survival time in palladin-overexpressing samples (Supplementary Table S1).

To account for the presence of normal, tumor-adjacent tissue (NAT) in the TCGA cohorts, we reanalyzed the TCGA-GBMLGG dataset with respect to the origin of the sample. Analysis of all 1130 samples (H_Kruskal–Wallis_ = 275.5, *p* < 0.0001, Supplementary Figure S1) revealed that, compared to healthy donor brain tissue, both primary and recurrent glioma tumors expressed roughly twice as much palladin (*p* < 0.0001 and *p* < 0.0001, respectively). A similar result was observed when comparing NAT to primary and recurrent glioma tumors (*p* = 0.0052 and *p* = 0.0042, respectively). Overexpression of PALLD in glioma tumors compared to non-tumor samples was also validated in six of the seven datasets containing non-tumor samples in the GlioVis website (Supplementary Figure S2).

### 3.2. Palladin Isoform 4 Is Specifically Overexpressed in Adult Glioma Tumors

To investigate the origin of palladin’s overexpression in glioma tumors, we analyzed data of various transcript expression levels in 1830 healthy and tumor samples. Overall, the PALLD gene has 18 transcripts, of which five are non-coding. Of the remaining 13, only four contain complete reading frames (Figure 1C). From analysis of the transcription patterns, we concluded that ENST00000505667, the canonical Ensembl palladin transcript, was not expressed in healthy brain tissue or in LGG or HGG tumors (H_Kruskal–Wallis_ = 167.6, *p* < 0.0001, Figure 1D). ENST00000261509 (H_Kruskal–Wallis_ = 537.3, *p* < 0.0001) doubled its transcription in LGG and HGG tumors compared to normal tissue (*p* < 0.0001 and *p* < 0.0001, respectively). A slight decrease in expression was seen from the LGG to HGG (*p* = 0.0283). ENST00000512127 (H_Kruskal–Wallis_ = 804.3, *p* < 0.0001) was not expressed in healthy brains or LGGs but was significantly more expressed in HGGs (*p* < 0.0001 and *p* < 0.0001, respectively). ENST00000507735 (H_Kruskal–Wallis_ = 432.7, *p* < 0.0001), also known as palladin 4 or the 90 kDa isoform, nearly doubled its expression from healthy tissue to LGG tumors and doubled again from LGG to HGG tumors (*p* < 0.0001 and *p* < 0.0001, respectively).

### 3.3. Somatic Mutations in Palladin’s Genomic Sequence Are Extremely Rare

To assess the prevalence and characteristics of somatic mutations in the PALLD gene in glioma tumors, we analyzed the TCGA-GBMLGG dataset. Of the 1154 samples in the dataset, 826 had data on genomic variation. Mutations in palladin’s genomic sequence were identified in only six samples (Figure 1C). These mutations included one 5′ untranslated region (UTR) mutation, two in-frame missense mutations, and one premature stop codon insertion. Two other tumors had a mixture of silent and missense mutations. Due to the low prevalence of palladin mutations in gliomas, we were unable to elucidate the impact of mutations in specific domains or the presence of mutations in general.

### 3.4. Palladin Is Not Overexpressed in Pediatric Glioma Tumors

To expand our investigation into palladin’s role in glioma tumors, we analyzed pediatric glioma datasets from the GlioVis website. Nine of the 24 datasets available contained relevant data and were used. Ultimately, we did not observe differences tying palladin to any specific clinical feature. Data published by Henriquez et al. provided insight on the differences in palladin expression between fetal and adult human brains. Eight fetal and eight adult brain samples were obtained from the frontal lobe and cerebellum of donors. An expression analysis of samples revealed significantly higher average PALLD expression in fetal than in adult brains (t_unpaired_ = 5.042, df = 14, *p* = 0.0002, Figure 1E).

### 3.5. Palladin Expression Is Confined Predominantly to the Cancerous Tissue in the Brain

**Figure 2:**
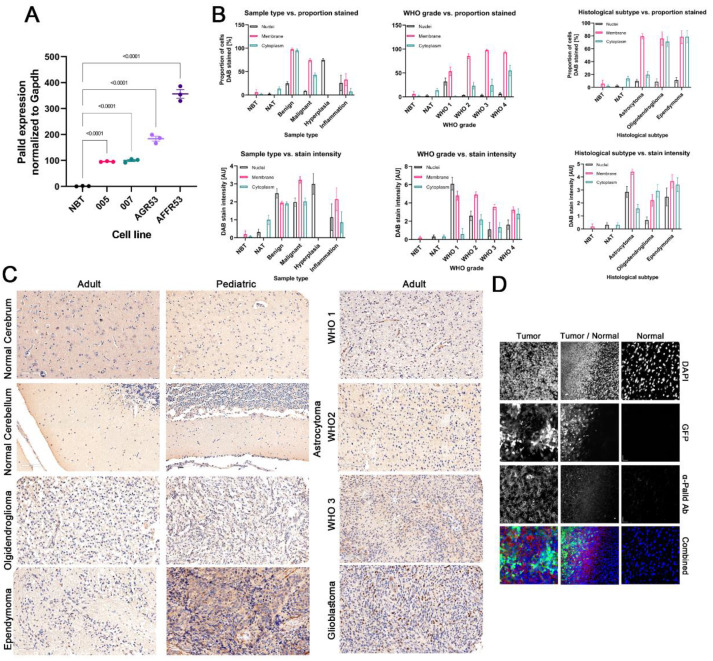
Validation of palladin expression in glioma cell lines and tissues (**A**) QRT-PCR analysis of palladin expression in murine glioblastoma cells and normal brain tissue. (**B**) Analysis of tissue microarray stained with an α-palladin antibody. Staining is shown in respect to sample type (left column), histological grade (middle column), and histological subtype (right column). Proportion (top row) and intensity (bottom row) are shown of stained nuclei (black), membrane (red), and cytoplasm (green). (**C**) Representative ×20 images of the tissue microarray from (**B**). (**D**) Representative ×63 images of α-palladin antibody-stained murine tumor and normal brain tissue. The data are shown as (**A**) means ±SEM. (**A**) One-way ANOVA with Holm–Šídák’s multiple comparisons test.

To validate our findings, we isolated total RNA from several mouse glioblastoma cell lines and normal brain tissue (NBT). In qRT-PCR, palladin was more expressed in glioblastoma cell lines than in NBT (F = 235.3, *p* < 0.0001, Figure 2A). We obtained a TMA with over 200 tissue cores of various CNS pathologies. Slides were immunohistochemically (IHC) stained with an anti-palladin antibody. The survey of the slide elucidated palladin’s presence in cell membranes, the cytoplasm, and the nuclei in the CNS. Healthy brain tissue and NAT exhibited weak to no staining in all three cellular compartments (Figure 2B,C and Supplementary Table S3). Benign tumors exhibited weak to moderate membrane and cytoplasmic staining in all surveyed cells and in the nuclei of 25% of the cells. In cores featuring glial hyperplasia, weak to moderate staining exclusively in the nucleus was seen in 75% of the cells. Weak to moderate staining in all three compartments was identified in 25% or less of the cells in CNS inflammation tissue cores. Malignant tumors exhibited weak to moderate membrane and cytoplasmic staining in 75% and 50% of cells, respectively. In approximately 15% of the malignant tumor cells, weak nuclear staining was observed.

Staining was analyzed with respect to WHO CNS tumor grade. Strong nuclear staining was seen almost exclusively in WHO CNS grade 1 tumors. The prevalence of membrane and cytoplasmic staining increased concordantly with tumor grade but did not surpass intensity levels of moderate and weak, respectively.

Weak to moderate nuclear staining was observed in ~8.5–11.3% of cells in astrocytomas, oligodendrogliomas, and ependymomas. In contrast, weak to moderate membranal staining was seen in over 75% of the cells evaluated in all three tumor subtypes. Finally, weak staining of the cell membrane was observed in most oligodendroglioma and ependymoma tumors, while the same intensity was seen in only 25% of astrocytoma tumors. In the vast majority of the cores, weak staining in the neuropil was seen.

We inspected palladin distribution in the area of contact between the tumor and NBT. We injected into mice brains glioblastoma cells that stably expressed GFP. Images showed clear localization of palladin staining to the area of cancer cells and not to the healthy tissue (Figure 2D lower row and upper row, respectively). The immunofluorescent staining images highlighted the localization of palladin to the membrane, but in contrast to the IHC images, they did not show staining in the tumor cell nucleus or cytoplasm. Palladin immunofluorescence staining extended faintly, slightly beyond the boundary layer of labeled tumor cells (Figure 2D middle row).

### 3.6. Glioblastoma Tumors Are Characterized by the Highest Levels of Palladin Expression

To deepen our understanding of the role of palladin in LGG tumors, we analyzed 525 non-GBM tumor samples grouped by the dataset’s original histopathologic type: astrocytomas, oligoastrocytomas, or oligodendrogliomas. PALLD mRNA levels differed significantly between tumor types (H_Kruskal–Wallis_ = 90.08, *p* < 0.0001, Supplementary Figure S3A). The highest levels of PALLD expression were found in astrocytoma tumors, followed by oligoastrocytoma, and, finally, oligodendroglioma tumors. Next, we analyzed the 5-year overall survival of LGG tumors grouped into three tertiles, based on palladin expression. Astrocytoma tumors exhibited a significant negative correlation between palladin expression and survival time (χ^2^_Mental-Cox_ = 13.1, df = 2, *p* = 0.0014, Supplementary Figure S4, top row). Median survival in astrocytomas featuring high, medium, and low levels of PALLD expression were 814, 1547, and 1339 days, respectively. Survival in oligodendroglioma and oligoastrocytoma tumors presented similar, albeit weaker (χ^2^_Mental-Cox_ = 6.06, df = 2, *p* = 0.0483, χ^2^_Mental-Cox_ = 4.468, df = 2, *p* = 0.1071, respectively), negative correlations to palladin expression.

**Figure 3:**
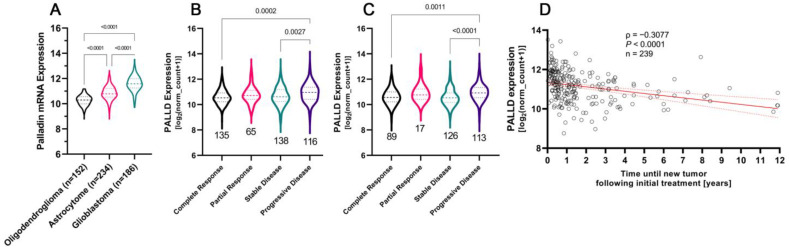
Aggressive gliomas in general, and glioblastoma tumor specifically, are characterized by higher levels of palladin. (**A**) Analysis of palladin expression in adult-type diffuse gliomas. (**B**,**C**) Palladin expression with respect to treatment outcome following primary and follow-up treatment. (**D**) Palladin expression levels plotted against the time until glioma recurrence following treatment. (**A**–**C**) The data are shown as the median with the first and third quartiles. (**A**) Kruskal–Wallis with Dunn’s multiple comparison test, (**B**,**C**) One-way ANOVA with Tukey multiple comparison tests, (**D**) Pearson correlation test, simple regression line in red with 95% confidence interval in gray.

We then reanalyzed the TCGA-GBMLGG dataset using the new WHO CNS5 classification. Overall, we identified 152 IDH-mutant and 1p/19q-codeleted oligodendroglioma tumors, 234 IDH-mutant astrocytoma tumors, and 186 IDH-wildtype glioblastoma tumors. We observed significant differences in mean palladin expression levels between tumors, according to the new classifications (H_Kruskal–Wallis_ = 287.5, *p* < 0.0001, Figure 3A). Glioblastoma tumors expressed 150% and 70% more palladin than oligodendroglioma and astrocytoma tumors, respectively (*p* < 0.0001 for both). Analysis of the 5-year overall survival according to the WHO CNS5 classification did not reveal significant variation that was dependent on palladin, within any tumor type (Supplementary Figure S4, bottom row).

### 3.7. Aggressive Glioma Tumors Are Characterized by Higher Levels of Palladin

To determine whether palladin expression correlates with tumor progression or response to treatment, we analyzed clinical and histopathologic data. We observed palladin overexpression in tumors presenting with progressive disease compared to stable disease, and compared to complete remission following primary treatment (F = 6.825, *p* = 0.0002, *p* = 0.0027, and *p* = 0.0002, respectively, Figure 3B). We noticed similar results in tumors after follow-up treatment (F = 8.692, *p* < 0.0001, *p* < 0.0001, and *p* = 0.0011, respectively, Figure 3C). Plotting the time to tumor recurrence against palladin expression produced a significant negative correlation (r_Pearson_ = −0.3077, *p* < 0.0001, Figure 3D). This indicated that more rapidly recurring tumors express higher levels of palladin. Further validation of palladin’s role in tumor aggression was obtained by analyzing the level of palladin expression in newly classified astrocytoma tumors across WHO CNS5 grades. We observed a clear increase in palladin transcription as the grade increased; WHO CNS5 grade 4 samples expressed 40% more palladin than WHO CNS5 grade 2 tumors (H_Kruskal–Wallis_ = 8.304, *p* = 0.0157, *P_Adj_* = 0.0217, Supplementary Figure S3B).

### 3.8. Palladin Expression Compared to Commonly Used Diagnostic and Prognostic Markers

**Figure 4:**
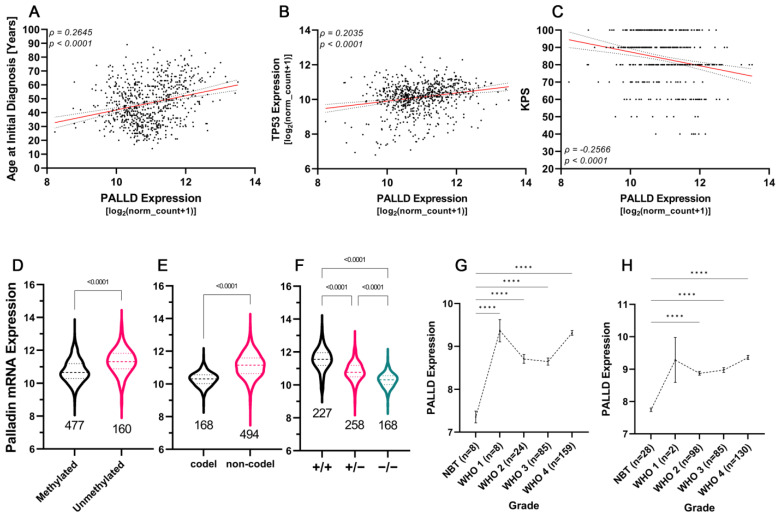
Palladin is a potential diagnostic and prognostic marker of glioma tumors. (**A**–**C**) Correlations of palladin expression with patient age at diagnosis, TP53 expression, and Karnofsky performance score, respectively. (**D**–**F**) Analysis of palladin expression with respect to MGMT promoter status, chromosome 1p/19q codeletion status, and IDH1 deletion status, respectively. (**G**,**H**) Palladin transcription levels in normal brain tissue and glioma tumors, ranging from WHO CNS grades 1–4. (**D**–**F**) The data are shown as the median with the first and third quartiles. (**G**,**H**) means ± SEM. (**A**–**C**) Pearson correlation test, simple regression line in red with 95% confidence interval in gray (**D**,**E**) Mann–Whitney test, (**F**) Kruskal–Wallis with Dunn’s multiple comparison test, (**G**) One-way ANOVA with Holm–Šídák’s multiple comparisons test (**H**) Brown–Forsythe and Welch ANOVA with Dunnett’s T3 multiple comparison tests, **** *p* < 0.0001.

Our next goal was to compare the expression of palladin and its predictive value, to established diagnostic and prognostic markers of glioma. We first plotted palladin expression against patient age, KPS, and TP53 expression. PALLD expression was significantly positively correlated with age at diagnosis and TP53 levels (r_Spearman_ = 0.2645, *n* = 695, *p* < 0.0001 and r_Spearman_ = 0.2035, *n* = 702, *p* < 0.0001, respectively, Figure 4A,B). Palladin levels were inversely correlated with KPS (r_Spearman_ = −0.2566, *n* = 439, *p* < 0.0001, Figure 4C). We then compared PALLD expression levels of samples with and without MGMT promoter methylation, and with the presence or absence of a 1p/19q chromosomal codeletion. Palladin was significantly overexpressed in tumors with an unmethylated MGMT promoter (U_Mann–Whitney_ = 21063, *p* < 0.0001, Figure 4D). Similarly, tumors with 1p/19q codeletion exhibited an almost two-fold increase in palladin expression compared to non-codeletion samples (U_Mann–Whitney_ = 12362, *p* < 0.0001, Figure 4E). Furthermore, palladin expression increased as IDH1 alleles were lost (H_Kruskal–Wallis_ = 291.1, *p* < 0.0001, Figure 4F), and reached a level double that of the baseline when both alleles were lost. Lastly, we fitted a multivariate Cox regression model to the overall survival data using the current clinical markers of glioma prognosis available in the TCGA-GBMLGG dataset, with and without palladin expression as a covariate. A naive model that included patient age, KPS, TP53 expression, and the presence of IDH1 mutation yielded a concordance score of 0.874 (Table 2). The presence of the IDH1 mutation was the most predictive covariate, with a hazard ratio (HR) of 0.1285 (*p* = 0.000933, 95% CI = 0.03814 to 0.4331). A model including palladin mRNA expression was then fitted to the survival data; this produced a 0.92 concordance score statistic (Table 3). PALLD expression (*p* = 0.00365, 95% CI = 1.518 to 8.548) and age at diagnosis (*p* = 0.01083, 95% CI = 1.016 to 1.134) proved the most influential markers for patient survival, with HRs of 3.6023 and 1.0736, respectively. Using a likelihood ratio test to compare the two models, the model incorporating palladin proved superior (χ^2^_likelihood ratio test_ = 9.7542, df = 1, *p* = 0.001789, Table 4). A third model was fitted with only the significant covariates, but this model performed worse than the other two, as the concordance score was only 0.852 (Supplementary Table S4). To examine whether palladin could be used in the diagnosis or risk stratification of glioma tumors, we analyzed all available datasets that included data about palladin expression, histological classification, and grade in non-tumor, LGG, and HGG samples. Overall, we analyzed 2638 samples from one pediatric and four adult datasets. Of the five datasets, only three included samples of WHO CNS grade 1 tumors, which were crucial for this type of analysis. In all three datasets, PALLD expression was significantly greater in tumor than non-tumor samples. In two of the datasets, PALLD expression was significantly greater in WHO CNS grade 1 tumors than in NBT (Figure 4G–H); while in the third dataset, PALLD expression presented with an obvious trend (Supplementary Figure S5).

### 3.9. Palladin Is Overexpressed Principally in Malignant Cells and Not in Other Glioma-Related Cell Types

**Figure 5:**
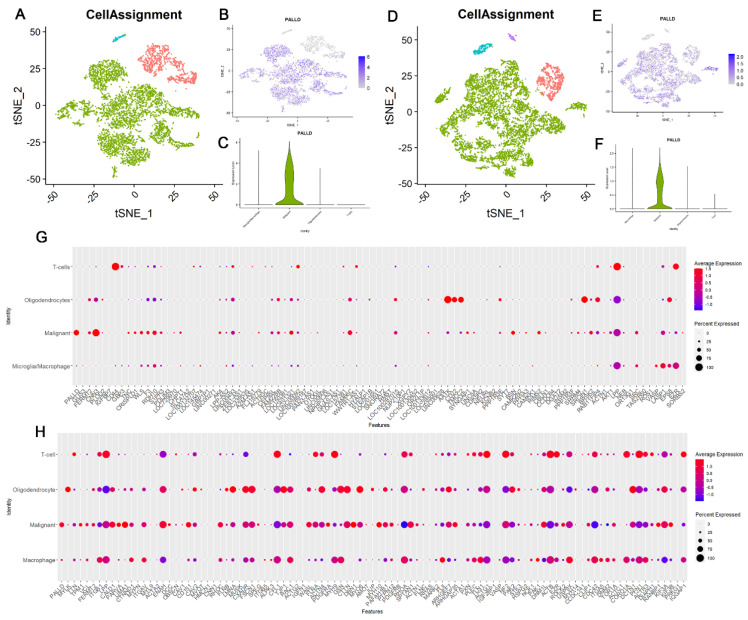
Palladin is uniquely expressed in the malignant cell population of glioma tumors. (**A**,**B**) T-distributed stochastic neighbor embedding (t-SNE) scatter plots of single-cell RNA sequencing (scRNAseq) of astrocytoma data, colored according to cell type and palladin expression, respectively. (**C**) Quantification of palladin expression in a number of cell types in astrocytoma scRNAseq data. (**D**,**E**) t-SNE scatter plots of scRNAseq of glioblastoma multiforme (GBM) data, colored according to cell type and palladin expression. (**F**) Quantification of palladin expression in a number of cell types in GBM scRNAseq data. (**G**,**H**) Expression quantification of 100 genes similar to palladin in a number of cell types in astrocytoma and GBM scRNAseq data, respectively. (**A**,**C**,**D**,**F**) Malignant cells are colored in green, oligodendrocytes in teal, T-cells in purple, and microglia/macrophages in red.

To examine the origin of palladin expression in glioma tumors, we obtained and analyzed single-cell RNA sequencing data of astrocytoma and GBM tumors. We analyzed 6225 cells originating from 10 IDH-mutant astrocytoma tumors designated as one of four cell types: malignant cells, microglia/macrophages, oligodendrocytes, or T cells. Palladin expression was detected in 71.6% of the malignant cells, 11.8% of microglia/macrophages, and 6.1% of oligodendrocytes (Figure 5A–C). In these respective samples, PALLD expression was 1.65, 0.18, and 0.09 times the mean level of the entire cohort. No palladin mRNA was identified in the T cell population. Next, we analyzed 7930 cells with identical cell classes in 28 GBM tumors. Palladin was observed in 62.2% of the malignant cells, 24.7% of microglia/macrophages, 4.6% of oligodendrocytes, and 1.1% of T cells (Figure 5D–F). In these respective samples, PALLD levels were 1.36, 0.56, 0.07, and 0.01 times the mean PALLD level of the entire cohort.

**Figure 6:**
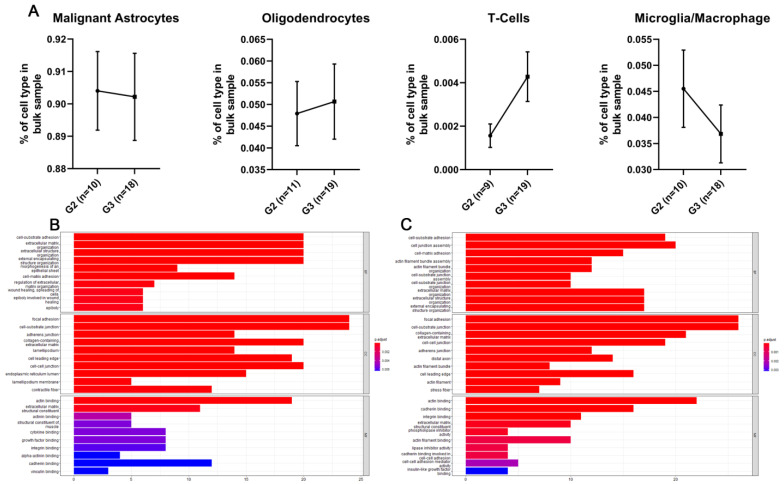
Palladin is overexpressed in malignant astrocytes and is involved in actin-based cellular motility. (**A**) In-silico flow cytometry of grades 2–3 IDH1-mutant astrocytoma tumors for malignant astrocytes, oligodendrocytes, T-cells, and microglia/macrophages (left to right). No significant changes are observed in cell population proportions as the tumor progresses. (**B**,**C**) Enrichment analysis of Gene Ontology terms using genes significantly co-expressed with palladin in astrocytoma and glioblastoma multiforme datasets, respectively. Many motility-related terms are observed. (**A**–**C**) The data are presented as mean ± SEM. (**A**–**C**) Mann–Whitney test.

As cytoskeletal genes, such as palladin, have important roles in many cellular functions, we compared palladin to other similar genes, to determine whether its pattern of expression is specifically associated with gliomas. We acquired a list of 100 genes that are similar to palladin using Gene Card Suite’s Genes Like Me algorithm. The similarity score was based on the relatedness between two genes, of their domains, sequence paralogy, expression patterns, modulating compounds, super pathways, phenotypes, Gene Ontology terms, and associated disorders. For the two studies included in our single-cell analysis, data were available for 98 of the 100 genes. We filtered the genes by whether their mean expression and proportion of expressing cells in the malignant cell population were equal to, or higher than, in PALLD (Figure 5G,H, Supplementary Tables S5 and S6). We then examined whether the pattern of expression of each of the filtered genes was unique to the malignant cell population (i.e., not found in the other cell types). Five of the filtered genes passed this criterion in both studies: LIMA1, WASL, DBN1, MYH10, and SPTAN1. These genes all mimic palladin’s single-cell expression pattern (Supplementary Figure S6). We analyzed the impact of these gene expression levels on the overall survival of adults with glioma in the TCGA-GBMLGG cohort. We found that LIMA1 and WASL showed no significant effect on overall survival, while DBN1, MYH10, and SPTAN1 all increased survival, with a concordant increase in their expression. Finally, we used in-silico flow cytometry to investigate if the increase in palladin expression might originate from changes in various cell population proportions within the tumor as it progresses. Analysis of cell proportions in grades II and III IDH1-mutated astrocytoma tumors (*n* = 32), using single cell and bulk RNAseq data, revealed no significant changes in the proportions of malignant astrocytes, macrophages, oligodendrocytes, and T-cells within a tumor, between grades II and III (Figure 6A).

### 3.10. Palladin Is Related to a Transcriptional Program Involved in Cellular Motility and the Extracellular Matrix

To gain insight into palladin’s potential role in transcriptional regulation, we analyzed gene co-expression with PALLD using the SEEK [Human] server. A total of 40 datasets were used for the co-expression analysis in astrocytoma tumors and 100 datasets were used for GBM. In the astrocytoma datasets, 193 significantly (α = 0.01) co-expressed genes were identified, while 155 genes were found in GBM datasets. Overrepresentation analysis of Gene Ontology terms from a search of the significant genes yielded terms related to the extracellular matrix, actin cytoskeleton, and cellular motility (Figure 6B,C).

## 4. Discussion

A previous study conducted in our laboratory identified palladin as a driver of breast cancer metastasis [37]. In the current work, we set out to identify other cancers in which palladin may play a promoting role. Our expression analysis of 25 organs revealed significant upregulation of palladin in bile duct, brain, breast, liver, lung, pancreas, stomach, and thyroid cancers; and downregulation in tumors of the bladder, cervix, colon, endometrium, esophagus, ovary, prostate, rectum, skin, testis, and uterus. These results align with numerous studies that described overexpression of palladin in pancreatic and breast cancers [32,33]. Cancers of muscular organs and of organs rich in muscle tissue (e.g., the endometrium and colon, respectively) exhibited decreased palladin expression. This is interesting, as several publications implicate palladin as an important player in the differentiation and maturation process of healthy muscle cells [25,50,51]. Similarly, bladder and testicular cancers have exhibited decreased palladin levels relative to healthy tissues in which palladin has a functional role [52,53,54]. This can be explained by changing transcription patterns or relative proportions of palladin-expressing cells within a tumor as it progresses.

To narrow our search to tumors that might be affected by palladin expression in a clinically relevant manner, we performed an overall survival analysis. Palladin expression was tightly correlated only with the overall survival of individuals with glioma. This result is surprising, as we expected associations of palladin with the survival of individuals with breast and pancreatic cancers, as palladin is known to contribute to aggressive tumor behavior by promoting cell invasion in these cancers. Palladin was shown to be essential in the morphology of reactive astrocytes [39,55], which contribute to the progression of glioma tumors. While this may partially explain palladin’s link to gliomas, research into palladin’s function in the CNS in general, and in glioma pathogenesis specifically, is too limited to conclude that this is the only reason. Analysis of gene expression at the isoform level revealed that the 90 kDa palladin transcript 4 was most abundant in healthy brain tissue, and that its expression increased concordantly with the grade of the tumors analyzed. This suggests that palladin’s role in glioma tumors is related to its F-actin bundling capabilities, mediated by immunoglobulin (Ig) tandem domains 3 and 4, which are present in this isoform [30,31]. Palladin may also act indirectly in gliomas via interactions with its binding partners. Additional experiments that knockdown palladin in vitro are required to verify these assumptions.

Our search for somatic mutations in palladin’s sequence did not reveal mutations in the vast majority of glioma tumors. This suggests that non-mutated palladin is crucial for cellular function and tumor development. This is consistent with publications describing the importance of palladin in maintaining proper cellular shape, motility and invasiveness, cell division, and embryonic development [18,20,32,33,39,52]. As the WHO CNS5 classification [2] considers pediatric and adult gliomas as distinct pathological types, we analyzed the patterns of palladin expression in pediatric, as well as adult, glioma datasets. Results from pediatric datasets did not confirm the results of adult gliomas. Palladin expression in fetal brains was significantly higher than in adult brains. This observation can explain the lack of a significant difference between tumor and healthy pediatric tissue, but this requires further validation.

Images of mouse tissues provided validation, at the protein level, of our findings of increased palladin mRNA levels in tumor compared to healthy brain tissue. We found a correlation between this increase and WHO CNS5 grade. Fluorescent imaging showed palladin’s localization to the site of glioblastoma cell injection in healthy mouse brain tissue. This finding was supported by qRT-PCR, which revealed higher palladin expression in glioblastoma cell cultures than in NBT. Palladin staining patterns appeared to include the neutrophil and membrane of tumor cells, as well as the cell edges. This raises the possibility of using palladin to delineate glioma tumors, similar to the use of delta-aminolevulinic acid in fluorescence-guided neurosurgery [56].

We then stained human TMA of CNS pathologies. The findings led us to conclude that while palladin is present in the nuclei and cytoplasm of cells in the CNS, its presence in the membrane is most indicative of pathology. Palladin’s proportion and intensity of membranal staining were greater in benign and malignant tumors than in healthy, hyperplastic, and inflamed tissues. In gliomas, the level of palladin detected in the membrane increased in correlation with the WHO CNS grade. This observation was not restricted to a specific tumor type. Our results also indicate that glioblastoma tumors are the tumors most prone to palladin overexpression. This raises the potential that palladin expression in glioma tumors can have diagnostic implications. Applying the original TCGA-GBMLGG tumor classification, we observed that survival of patients with astrocytomas seems to decrease as palladin levels increase, in a dose response manner. When adult glioma tumors were classified according to the updated WHO CNS5 guidelines, differences in 5-year survival were independent of palladin expression within each tumor type (but not across types). As WHO CNS5 emphasizes molecular aspects of CNS tumor diagnosis, we believe this result strengthens the potential use of palladin as a tool for molecular diagnosis and differentiation of adult gliomas. It appears that more than predicting the survival of patients, palladin accurately predicts tumor type, such that deadlier tumors express more palladin. Further research aimed at defining clear cutoff values in patients is still needed. To assess the potential for actual clinical applications of palladin, we examined the rapidity of the increase in palladin level within a tumor. Palladin expression appears to rise immediately when the tissue is transformed, and the tumor is classified as grade 1. This supports the potential of palladin expression as a diagnostic marker for glioma tumors.

We identified links between palladin expression levels, poor treatment response, and earlier recurrence. Consequently, we studied palladin’s potential as a prognostic marker. We showed a correlation of palladin to common prognostic features, such as patient age at diagnosis and TP53 expression, and an inverse correlation with KPS. Furthermore, we used the Cox multivariate regression to analyze palladin’s association with mortality. Our results indicate that the level of palladin expression is a stronger predictor, than the currently used prognostic markers, of the overall survival of individuals with glioma.

Whether palladin can be used in the treatment of glioma tumors is another matter of interest. A recent review by Torrisi et al. summarized the hallmarks of glioblastoma tumors and described them as highly invasive, though less prone to distant metastasis. Invasion is a complex process involving the loss of cellular adhesion, epithelial-to-mesenchymal transition, increased cell motility, and degradation and reorganization of the surrounding extracellular matrix. Governing this invasion are signaling pathways related to hypoxia and inflammation. Inflammation and subsequent myeloid cell recruitment increase the expression and signaling of transforming growth factor beta and platelet-derived growth factor [57]. These both lead to epithelial-to-mesenchymal transition and rely on palladin expression downstream [25,58,59,60]. The ability of hypoxia to trigger invasion in glioblastomas has been extensively studied. Several publications tie palladin to elements of the hypoxia-related PI3K/AKT/mTOR pathway [61,62]. In this regard, it is important to note reports describing the ability of AKT1 and 2 to modulate palladin f-actin binding activity and expression, respectively [63,64]. The SRC pathway is a proto-oncogene non-receptor tyrosine kinase pathway that is capable of remodeling the cytoskeleton in response to hypoxic stimuli [62]. Ronty et al. reported that SRC-mediated, platelet-derived growth factor-induced membrane ruffling and lamellipodia formation required both palladin and SPIN90 [58]. Taken together, it is reasonable to assume that downregulation of palladin or inhibition of its binding to the actin cytoskeleton might curb the aggressive phenotype of glioma tumors. Nevertheless, additional experimental work is needed to determine the actual clinical value of this.

To better understand the overexpression of palladin in gliomas, we analyzed scRNAseq data from astrocytoma and GBM tumors. Palladin expression appears to originate mostly from malignant cells. To validate our findings, we investigated the expression of 100 genes genetically, phenotypically, structurally, and transcriptionally similar to palladin. Our results reinforce the uniqueness and clinical relevance of palladin’s transcription patterns in glioma tumors.

## 5. Conclusions

To conclude, our findings suggest that overexpression of wild-type palladin’s isoform 4, originating mostly from the malignant cell population, is involved in the progression of aggressive adult glioma tumors, and that this expression correlates to decreased survival. Our results introduce the possibility of using palladin as a diagnostic and prognostic marker. Additional experimentation is needed to determine the potential use of palladin as a therapeutic target.

## Figures and Tables

**Table 1:** Summary of all the comparisons examined of palladin expression in tumor and healthy tissues from different organs.

Organ/Region	Study & Cohort	Statistic	*p*	PALLD in Tumor
Adrenal gland	TCGA-ACC + GTEx	U_Mann–Whitney_ = 4299	0.2364	→
Bile duct	TCGA-CHOL + GTEx	t_unpaired_ = 2.242, df = 43	0.0302	↑
Bladder	TCGA-BLCA + GTEx	t_unpaired_ = 8.511, df = 433	<0.0001	↓
Brain	TCGA-GBM + TCGA-LGG + GTEx	U_Mann–Whitney_ = 147,235	<0.0001	↑
Breast	TCGA-BRCA + GTEx	U_Mann–Whitney_ = 110,310	<0.0001	↑
Cervix	TCGA-CESC + GTEx	t_unpaired_ = 5.24, df = 306	<0.0001	↓
Colon	TCGA-COAD + GTEx	U_Mann–Whitney_ = 21,873	<0.0001	↓
Endometrium	TCGA-UCEC + GTEx	U_Mann–Whitney_ = 308	<0.0001	↓
Esophagus	TCGA-ESCA + GTEx	U_Mann–Whitney_ = 48,610	<0.0001	↓
Head & neck reion	TCGA-HNSC + GTEx	U_Mann–Whitney_ = 10,902	0.6338	→
Kidney	TCGA-KIRC + TCGA-KIRP + GTEx	U_Mann–Whitney_ = 81,033	0.2866	→
Liver	TCGA-LIHC + GTEx	U_Mann–Whitney_ = 14,504	<0.0001	↑
Lung	TCGA-LUAD + TCGA-LUSC + GTEx	U_Mann–Whitney_ = 182,850	0.014	↑
Ovary	TCGA-OV + GTEx	U_Mann–Whitney_ = 12,349	<0.0001	↓
Pancreas	TCGA-PAAD + GTEx	U_Mann–Whitney_ = 17.5	<0.0001	↑
Paraganglia	TCGA-PCPG + GTEx	t_unpaired_ = 0.7294, df = 183	0.4667	→
Prostate	TCGA-PRAD + GTEx	U_Mann–Whitney_ = 27,055	<0.0001	↓
Rectum	TCGA-READ + GTEx	t_Welch-corrected_ = 4.435, df = 9.872	0.0013	↓
Skin	TCGA-SKCM + GTEx	U_Mann–Whitney_ = 67,245	<0.0001	↓
Soft tissue & bone	TCGA-SARC + GTEx	U_Mann–Whitney_ = 215	0.6772	→
Stomach	TCGA-STAD + GTEx	U_Mann–Whitney_ = 37,554	0.0105	↑
Testis	TCGA-TGCT + GTEx	U_Mann–Whitney_ = 7660	<0.0001	↓
Thymus	TCGA-THYM + GTEx	U_Mann–Whitney_ = 45.5	0.1532	→
Thyroid	TCGA-THCA + GTEx	U_Mann–Whitney_ = 10,832	0.0028	↑
Uterus	TCGA-UCS + TCGA-UCEC + GTEx	t_Welch-corrected_ = 12.95, df = 85.67	<0.0001	↓

**Table 2:** Results of the multivariate Cox regression model—not including palladin.

Covariates	Coefficient	Standard Error	*p*	HR	Lower CI_95%_	Upper CI_95%_
TP53	0.42027	0.50473	0.405035	1.5224	0.5661	4.0939
Age at diagnosis	0.4777	0.0245	0.051199	1.0489	0.99975	1.1005
KPS score	−0.0442	0.03717	0.234326	0.9568	0.88954	1.0291
IDH1 mutation (0: no, 1: yes)	−2.05162	0.61982	0.000933	0.1285	0.03814	0.4331

HR = Hazard Ratio, CI = Confidence Interval, KPS = Karnofsky Performance Score.

**Table 3:** Results of the multivariate Cox regression model—including palladin.

Covariates	Coefficient	Standard Error	*p*	HR	Lower CI_95%_	Upper CI_95%_
PALLD	1.281586	0.440867	0.00365	3.6023	1.51817	8.548
TP53	0.846903	0.600697	0.15858	2.3324	0.71861	7.57
Age at diagnosis	0.070992	0.027862	0.01083	1.0736	1.01652	1.134
KPS score	−0.008419	0.040141	0.83388	0.9916	0.91659	1.073
IDH1 mutation (0: no, 1: yes)	−1.319920	0.680868	0.05255	0.2672	0.07034	1.015

HR = Hazard Ratio, CI = Confidence Interval.

**Table 4:** Results of the multivariate Cox regression model—including palladin.

DF	Log Likelihood	DF	χ^2^	*p*
4	−39.743			
5	−34.866	1	9.7542	0.001789

DF = Degrees of Freedom.

## Data Availability

Publicly available datasets were analyzed in this study. This data can be found here: https://xenabrowser.net/, http://gliovis.bioinfo.cnio.es/ and https://singlecell.broadinstitute.org/single_cell.

## Supplementary Materials

The following supporting information can be downloaded at: https://www.mdpi.com/article/10.3390/cancers14205130/s1, Figure S1. Reanalyzing palladin expression against sample type in the TCGA-GBMLGG dataset; Figure S2. Analysis of palladin expression relative to sample type in adult glioma datasets from the GlioVis server; Figure S3. Expression of palladin non-glioblastoma gliomas; Figure S4. Overall survival of patients with different types of glioma tumors; Figure S5. Palladin transcription levels in normal brain tissue (NBT) and glioma tumors in the Rembrandt dataset; Figure S6. Expression patterns of palladin similar genes in astrocytoma and GBM scRNAseq data; Table S1. Complete list of datasets used; Table S2; Table S3. Summary table of IHC results; Table S4. Results of the multivariate Cox regression model—only significant covariates; Table S5. Analysis of gene expression in scRNAseq astrocytoma dataset; Table S6. Analysis of gene expression in the scRNAseq GBM dataset.
