# Supplementary material for: Cytoskeletal Protein Palladin in Adult Gliomas Predicts Disease Incidence, Progression, and Prognosis
Source: Cancers (Basel). 2022 Oct 19;14(20):5130. doi: 10.3390/cancers14205130 (PMC9600953; doi:10.3390/cancers14205130)
Supplement: Supplementary file 1 [file cancers-14-05130-s001.zip › supp_figures_vfinal.pdf]

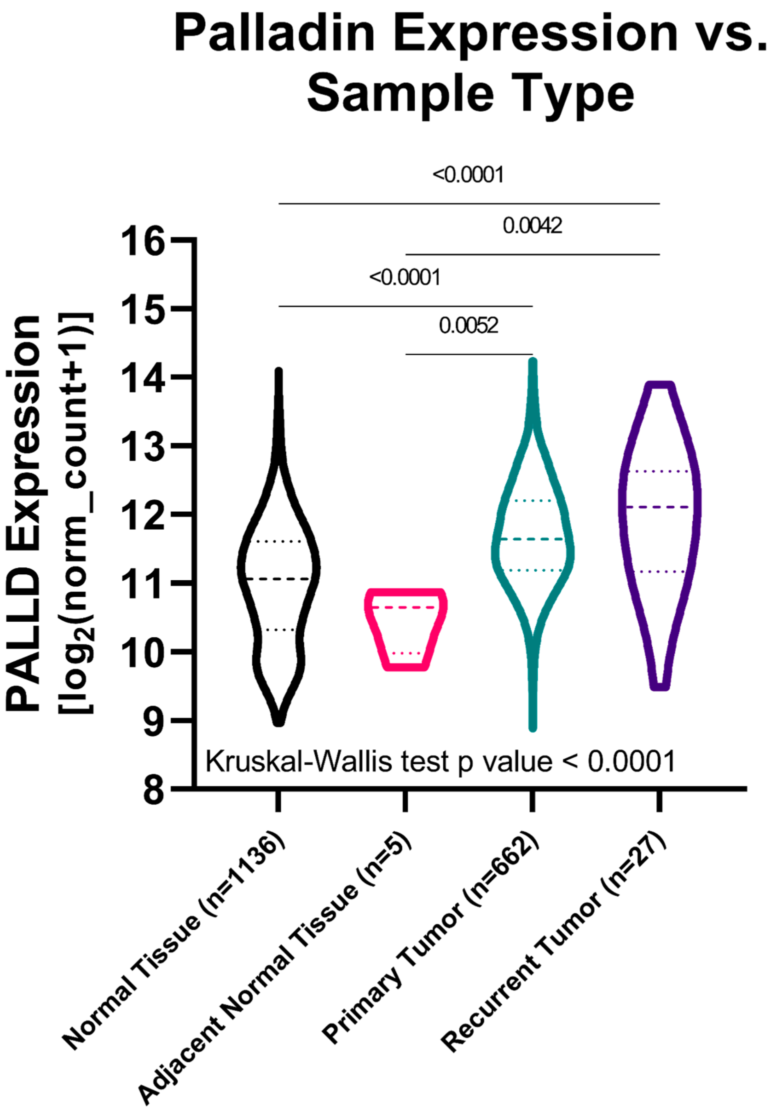

Supplementary Figure 1. Reanalyzing palladin expression against sample type in the TCGA-GBMLGG dataset. compared to healthy donor brain tissue, both primary and recurrent glioma tumors ex-pressed roughly twice as much palladin. The data are shown as the median with the first and third quartiles

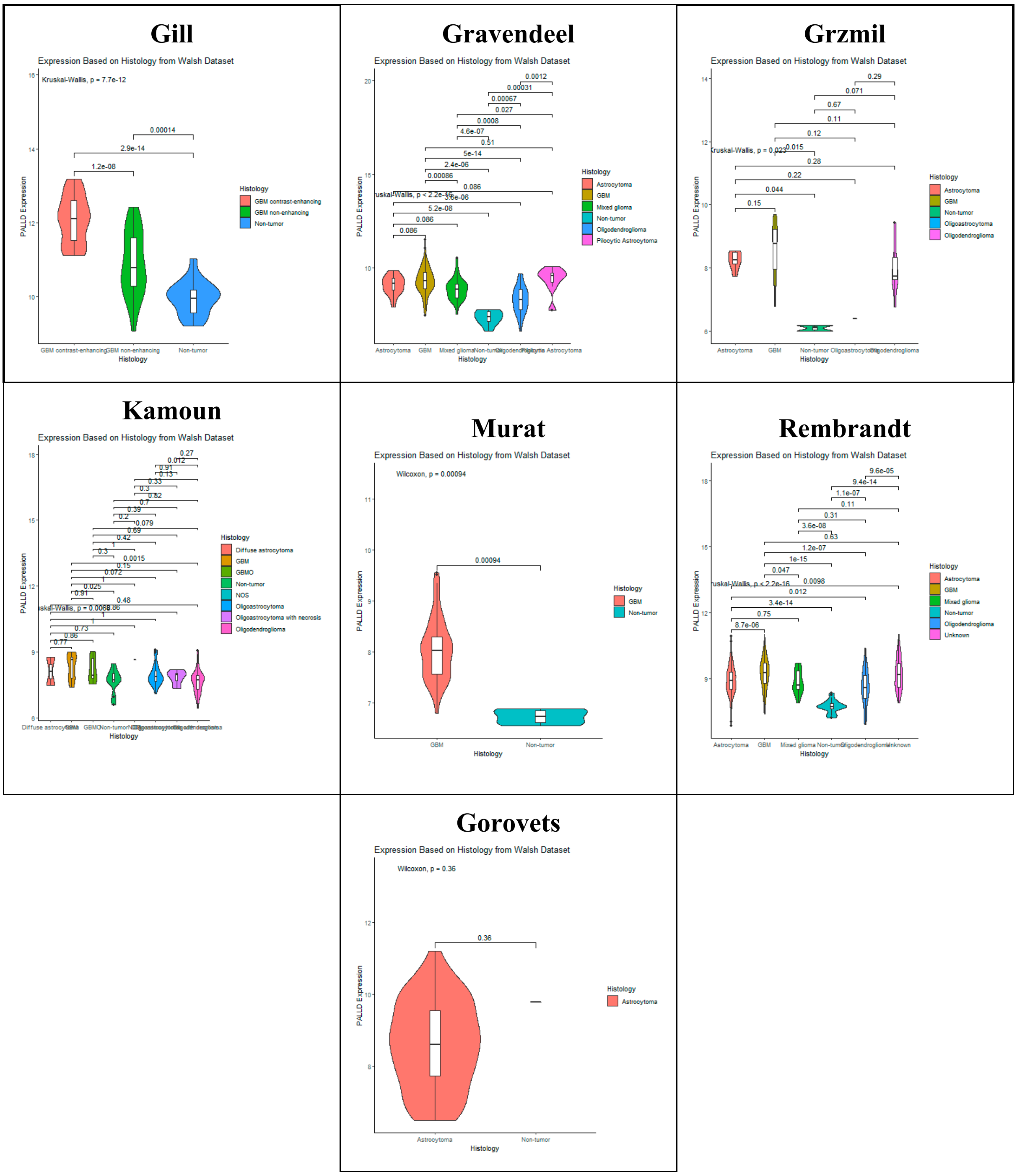

Supplementary Figure S2. Analysis of palladin expression relative to sample type in adult glioma datasets from the GlioVis server. Overexpression of PALLD in glioma tumors compared to non-tumor samples is observed in six of the seven datasets.

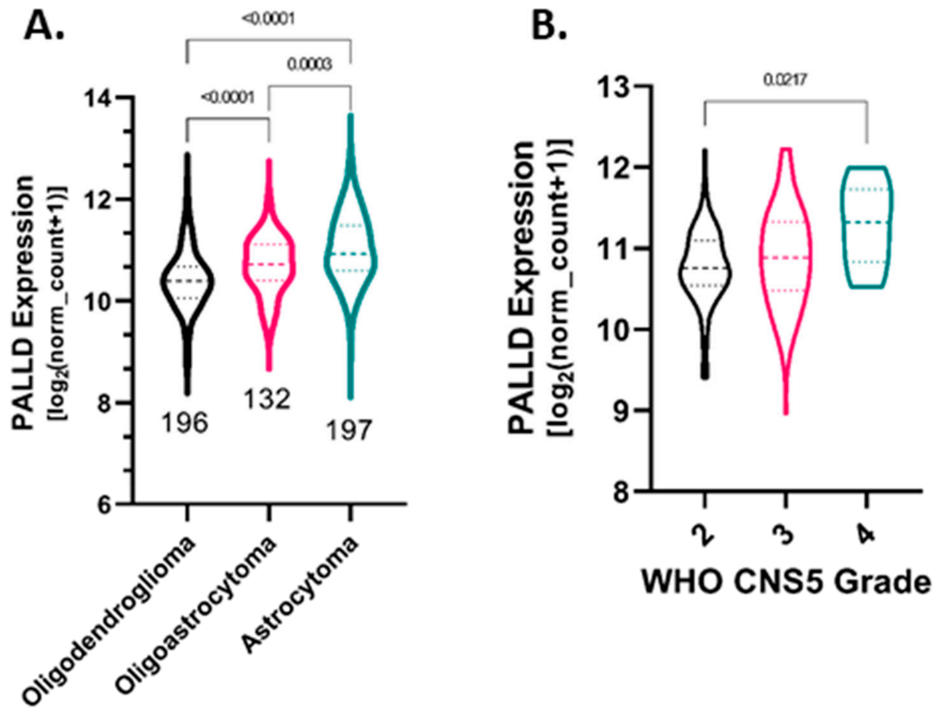

Supplementary Figure S3. Expression of palladin non-glioblastoma gliomas. (A) Analysis of palladin expression in different histological subtypes of lower grade glioma tumors. (B) Analysis of palladin expression in IDH-mutant astrocytoma tumors across different WHO CNS5 grades. The data are shown as the median with the first and third quartiles. (A-B) Kruskal-Wallis with Dunn's multiple comparison test.

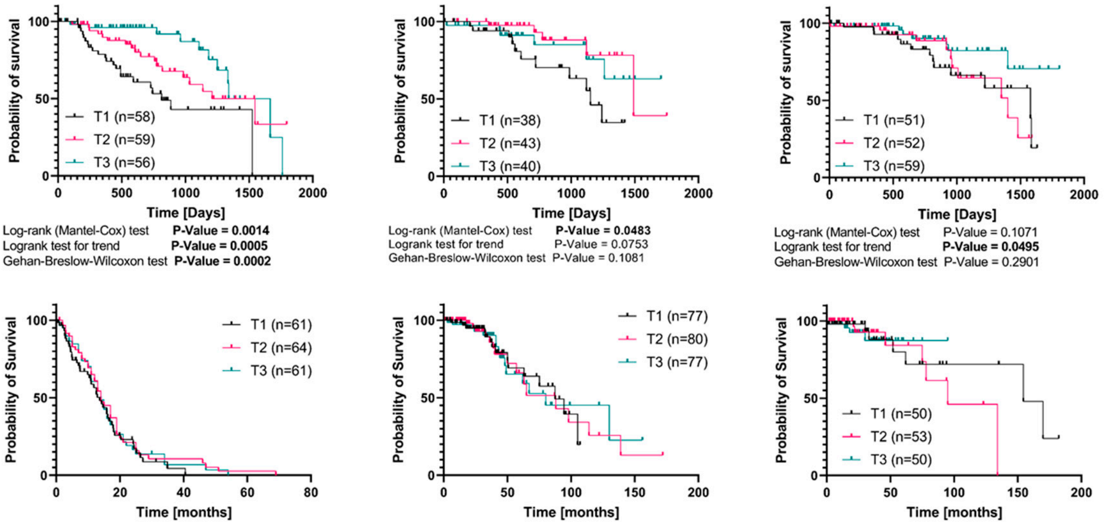

Supplementary Figure S4. Overall survival of patients with different types of glioma tumors. Overall survival of glioma patients using the original (and old) TCGA-GBMLGG glioma classification (top row) and the new WHO CNS5 classification (bottom row). Survival data were stratified into three groups based on palladin expression levels.

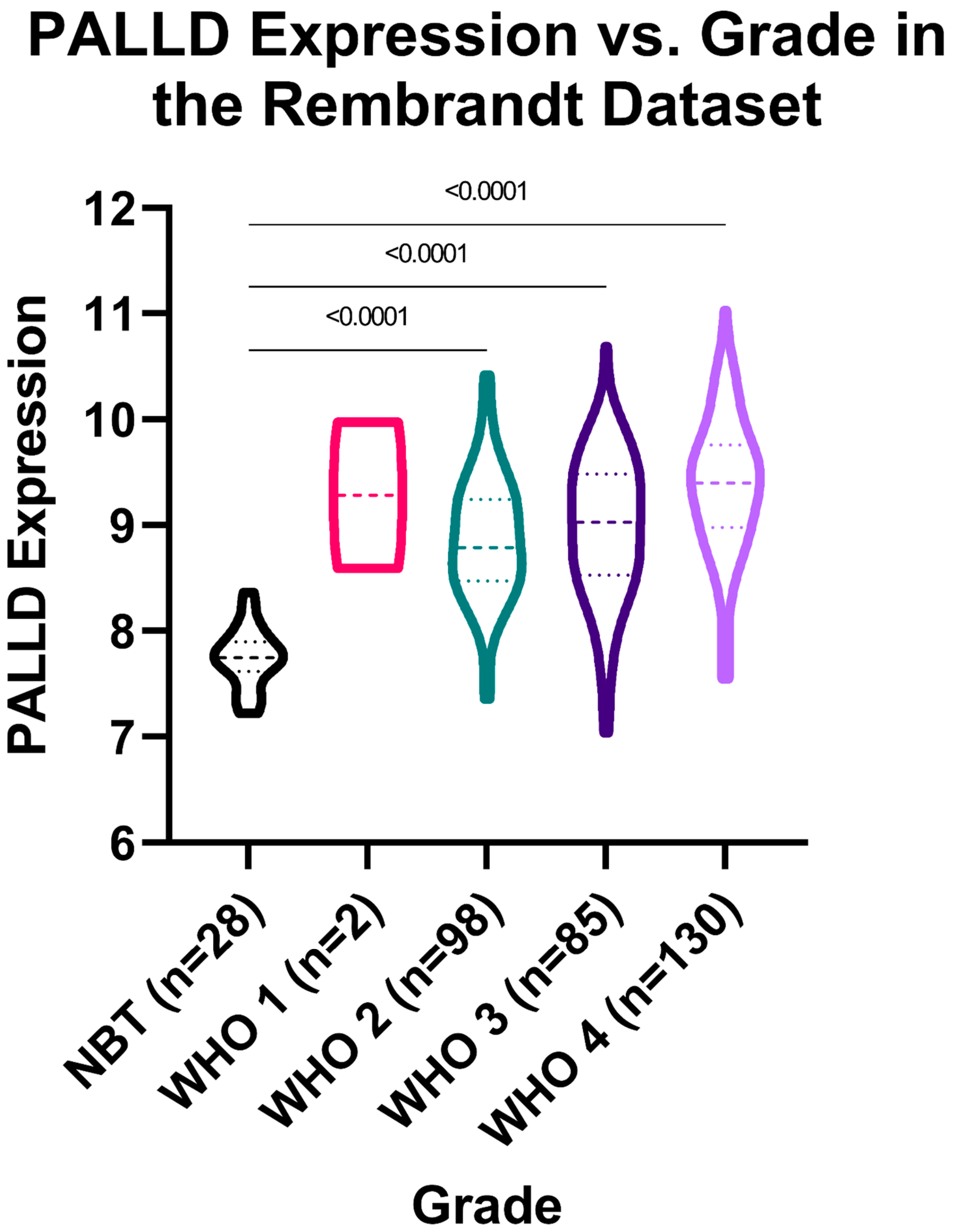

Supplementary Figure S5. Palladin transcription levels in normal brain tissue (NBT) and glioma tumors in the Rembrandt dataset. Palladin transcription increases with the WHO CNS grade as soon as the tissue is transformed.

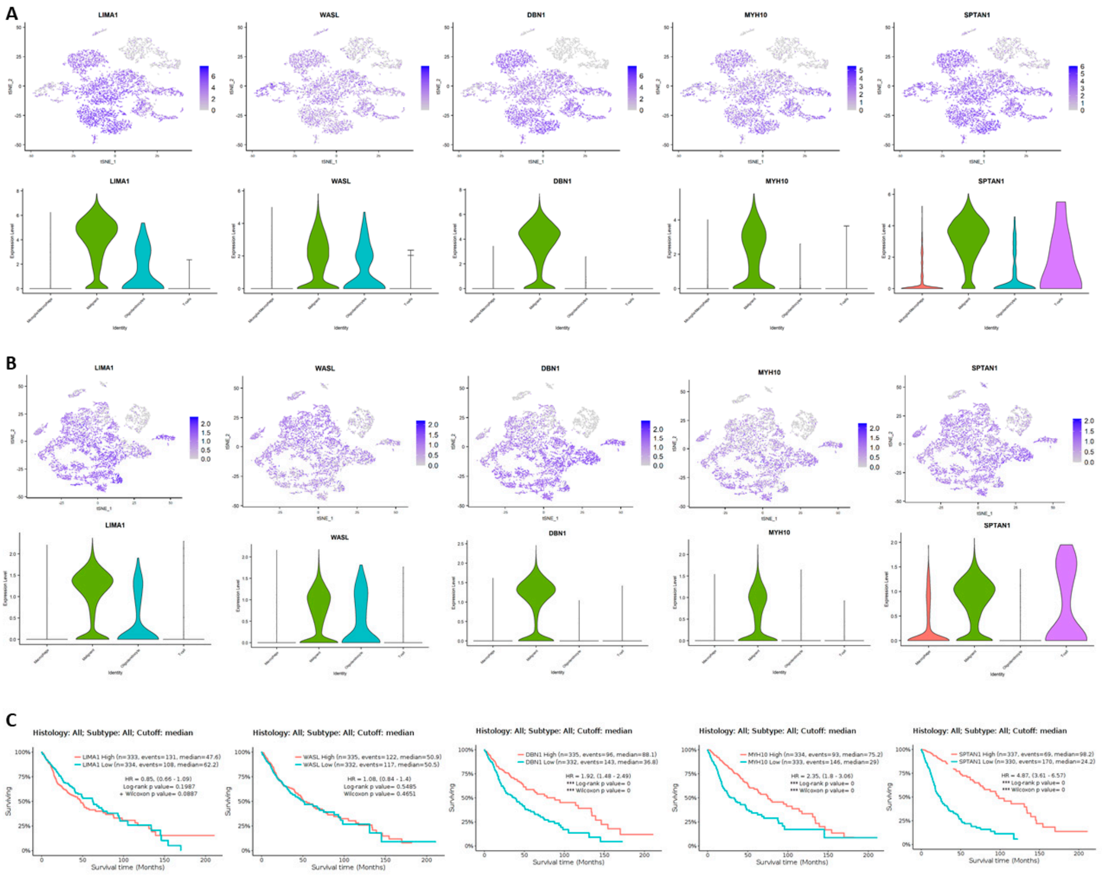

Supplementary Figure S6. Expression patterns of palladin similar genes in astrocytoma and GBM scRNAseq data. (A) t-distributed stochastic neighbor embedding, (t-SNE) scatter plots colored according to palladin expression (top row) and quantification of expression in different cell types (lower row) of the genes most similar to palladin in astrocytoma scRNAseq data. (B) t-SNE scatter plots colored according to palladin expression (top row) and quantification of expression in different cell types (lower row) of the genes most similar to palladin in GBM scRNAseq data. (C) Overall survival curves of individuals with glioma tumors from the TCGA-GBMLGG dataset. Survival data were divided into two groups based on the median of palladin expression levels.
