# Supplementary material for: Cytoskeletal Protein Palladin in Adult Gliomas Predicts Disease Incidence, Progression, and Prognosis
Source: Cancers (Basel). 2022 Oct 19;14(20):5130. doi: 10.3390/cancers14205130 (PMC9600953; doi:10.3390/cancers14205130)
Supplement: Supplementary file 1 [file cancers-14-05130-s001.zip › supp_tables_vfinal.pdf]

Supplementary Table 1. Complete list of datasets used.

Dataset	Data type	Samples			Available data				Used in our analysis?	Survival w/ PALLD OE?	Significant (α=0.05)?
		Non-tumor	Low-grade	GBM	Histology	Grade	Subtype	Survival			
CGGA	RNA-seq	0	625	388	✔	✔	✔	✔	✔	↓	Yes
TCGA GBM	HG-UG133A	10	0	528	✔	-	✔	✔	-	N/A	N/A
	Agilent-4502A	10	0	489	✔	-	✔	✔	-	N/A	N/A
	RNA-seq	4	0	156	✔	-	✔	✔	-	N/A	N/A
TCGA LGG	RNA-seq	0	513	0	✔	✔	✔	✔	-	N/A	N/A
TCGA GBMLGG	RNA-seq	0	515	152	✔	✔	✔	✔	✔	↓	Yes
Rembrandt	Microarray	28	225	219	✔	✔	✔	✔	✔	↓	Yes
Gravendeel	Microarray	8	117	159	✔	✔	✔	✔	✔	↓	Yes
Bao	RNA-seq	0	174	100	✔	-	✔	-	-	N/A	N/A
Kamoun	Microarray	9	154	16	✔	✔	✔	✔	✔	↓	Yes
Ivy GAP	RNA-seq	0	0	270	✔	-	✔	-	-	N/A	N/A
Lee Y	Microarray	0	0	191	-	-	✔	✔	✔	↑	Yes
Oh	Microarray	0	0	105	-	-	✔	-		N/A	N/A
Phillips	Microarray	0	24	76	✔	✔	✔	✔	✔	↓	Yes
Gill	RNA-seq	17	0	75	✔	-	✔	-	-	N/A	N/A
Freije	Microarray	0	26	59	✔	✔	✔	✔	✔	↓	Yes
Murat	Microarray	4	0	80	✔	-	✔	✔	✔	↓	No
Gorovets	Microarray	1	80	0	✔	✔	✔	✔	✔	↑	Yes
POLA Net.	Microarray	0	71	0	-	✔	✔	✔	✔	↓	Yes
Reifenberger	Microarray	0	0	70	-	-	✔	-	-	N/A	N/A
Joo	Microarray	0	0	57	-	-	✔	✔	✔	↑	Yes
Ducray	Microarray	0	0	52	-	-	✔	✔	✔	↑	No

Walsh	Microarray	0	0	52	-	-	✔	-	-	N/A	N/A
Kwom	Microarray	0	0	43	-	-	✔	-	-	N/A	N/A
Nutt	Microarray	0	22	28	✔	-	✔	✔	✔	↓	Yes
Vital	Microarray	0	12	26	✔	✔	✔	✔	✔	↑	Yes
Grzmil	Microarray	2	15	15	✔	-	✔	✔	✔	↓	Yes
Gleize	Microarray	0	30	0	✔	✔	-	-	-	N/A	N/A
Donson	Microarray	0	5	21	✔	-	✔	✔	✔	↑	No
Li	Microarray	0	0	23	-	-	✔	-	-	N/A	N/A

Supplementary Table 3. Summary table of IHC results

Table is too big to be pasted here.

Supplementary Table 4. Results of the multivariate Cox regression model - only significant covariates.

<table>
<tr><th rowspan="2" colspan="2">Covariates</th><th rowspan="2" colspan="2">Coefficient</th><th rowspan="2">Standard Error</th><th rowspan="2" colspan="2">P value</th><th rowspan="2">HR</th><th colspan="2">95% CI</th></tr>
<tr><th>Lower</th><th>Upper</th></tr>
<tr><td colspan="2">PALLD</td><td colspan="2">1.11599</td><td>0.32630</td><td colspan="2">0.000626</td><td>3.0526</td><td>1.6103</td><td>5.7866</td></tr>
<tr><td colspan="2">Age at diagnosis</td><td colspan="2">0.05188</td><td>0.02115</td><td colspan="2">0.014184</td><td>1.0532</td><td>1.0105</td><td>1.0978</td></tr>
<tr><td colspan="2">IDH1 mutation (0: no, 1: yes)</td><td colspan="2">-1.12182</td><td>0.55954</td><td colspan="2">0.044974</td><td>0.3257</td><td>0.1088</td><td>0.9752</td></tr>
<tr><td>Concordance</td><td colspan="2">0.852 (se = 0.047)</td><td>Likelihood ratio test</td><td colspan="2">28.52 on 3 df, P=3e-06</td><td>Wald test</td><td>25.76 on 3 df, P=1e-05</td><td>Score (logrank) test</td><td>36.44 on 3 df, P=6e-08</td></tr>
</table>

Supplementary Table 5. Analysis of gene expression in scRNAseq astrocytoma dataset

#	id/CLUSTER	Fraction Expressing Gene X				Scaled Mean Expression of Gene X			
		microglia/macrophage	malignant	oligodendrocytes	T cells	microglia/macrophage	malignant	oligodendrocytes	T cells

1	PALLD	11.84	71.59	6.12	0	0.18	1.65	0.09	0
2	MYLK	0.96	9.42	92.86	11.11	0.01	0.12	4.7	0.21
3	TTN	22.62	32.69	22.45	55.56	0.06	0.1	0.07	1.14
4	TPM1	16.07	36.71	8.16	0	0.47	0.97	0.16	0
5	VCL	27.82	26.05	10.2	11.11	0.54	0.48	0.07	0.58
6	FERMT2	1.73	38.45	36.73	0	0.02	1.03	1	0
7	ITGB1	75.94	67.47	70.41	55.56	2.65	1.95	2.45	2.31
8	LPP	98.56	98.9	100	100	1.31	1.52	0.98	2.25
9	CALD1	2.5	48.68	75.51	0	0.02	0.93	1.68	0
10	PARVA	13.86	65.9	16.33	0	0.02	1.26	0.06	0
11	LIMA1	15.98	89.95	48.98	11.11	0.39	3.91	1.37	0.26
12	CTNND1	53.22	53.09	48.98	11.11	0.58	0.55	0.54	0.03
13	MYPN	0.67	0.45	0	0	0	0	0	0
14	ITGAV	64.97	55.9	64.29	22.22	1.19	0.98	1.41	0.47
15	MYL9	4.04	0.39	1.02	0	0.09	0.01	0	0
16	ACTA2	14.44	2	2.04	22.22	0.44	0.03	0.01	0.52
17	ENAH	73.63	97.78	89.8	55.56	0.18	2.24	1.17	0.3
18	DCC	1.44	34.14	7.14	0	0	0.63	0.2	0
19	OBSCN	5.68	5.45	5.1	0	0.02	0.03	0.02	0

20	CAV1	1.06	3.1	13.27	0	0.01	0.09	0.34	0
21	CD151	33.11	53.48	10.2	0	0.67	1.04	0.23	0
22	CAV2	33.88	51.15	46.94	33.33	0.17	0.26	0.55	0.23
23	MYOT	1.06	7.73	32.65	0	0.01	0.21	0.84	0
24	HMCN1	2.12	3.49	2.04	0	0.01	0.02	0.01	0
25	CNN1	0.38	0.33	0	0	0.01	0.01	0	0
26	RHOC	45.72	61.27	13.27	11.11	1.7	2.46	0.36	0.14
27	FLNB	5	29.31	68.37	11.11	0.04	0.38	1.31	0.57
28	LMNA	37.63	50.54	94.9	0	1.34	1.19	4.49	0
29	MUSK	1.06	0.39	9.18	0	0.01	0	0.15	0
30	CXADR	58.23	79.01	86.73	66.67	0.8	1.47	3.51	0.47
31	FSCN1	84.7	80.75	86.73	0	2.27	1.64	2.46	0
32	SPEG	0.58	30.82	1.02	0	0	0.45	0	0
33	ITGB5	31.38	2.06	15.31	11.11	0.76	0.03	0.44	0.18
34	ALPK3	35.9	2	2.04	0	0.6	0.02	0.01	0
35	CFL1	99.52	99.86	100	100	6.36	6.2	6.34	6.77
36	TJP1	13.86	78.24	93.88	0	0.21	1.45	2.41	0
37	RAC1	96.25	91.88	87.76	33.33	4.05	3.15	3.05	0.75
38	ACTG2	1.44	1.59	2.04	11.11	0.01	0.01	0.01	0.11

39	IGFN1	0.29	0.71	0	0	0	0	0	0
40	WASL	24.45	74.28	50	22.22	0.42	1.72	1.05	0.49
41	FLNA	22.23	60.82	5.1	88.89	0.32	1.06	0.04	3.58
42	ACTN4	33.88	67.82	77.55	77.78	0.75	1.53	2.55	2.32
43	PDGFRA	2.31	81.77	8.16	22.22	0.01	3.47	0.02	0.2
44	MYH9	90.47	24.31	65.31	100	2.32	0.31	1.25	4.42
45	GSN	98.56	9.4	100	0	6.45	0.27	7.99	0
46	CTTN	6.16	92.86	100	0	0.05	3.7	4.82	0
47	DBN1	2.6	84.48	7.14	0	0.02	3.38	0.06	0
48	MYO6	29.64	71.81	91.84	22.22	0.32	1.36	2.98	0.26
49	AMOT	15.78	10.57	6.12	0	0.29	0.15	0.04	0
50	JUP	13.09	9.3	42.86	0	0.32	0.21	1.22	0
51	MYH10	18.77	78.44	14.29	11.11	0.14	1.93	0.06	0.41
52	PAFAH1B1	56.69	79.36	63.27	55.56	1.01	2.02	1.47	1.73
53	PTPN12	37.05	47.11	31.63	11.11	0.86	1.14	0.67	0.07
54	PDGFRB	1.25	5.24	1.02	0	0.01	0.1	0	0
55	RHOA	97.69	93	98.98	77.78	5.91	4.7	6.12	4.41
56	SPTAN1	33.11	95.64	33.67	55.56	0.5	3.09	0.68	1.49
57	ACTN1	6.45	13.71	4.08	0	0.14	0.31	0.01	0

58	FLNC	0.96	1.88	51.02	0	0.01	0.02	1.2	0
59	RRAS	9.24	2.22	3.06	0	0.31	0.07	0.12	0
60	MARK2	23.87	29.7	34.69	22.22	0.23	0.25	0.33	0.15
61	FLII	70.84	62.27	61.22	55.56	2.23	1.66	1.91	2.35
62	ARHGEF7	55.15	91.92	84.69	55.56	1.2	3.18	2.65	1.9
63	ARHGAP35	43.98	73.12	40.82	33.33	0.59	1.24	0.53	0.59
64	ACTA1	1.64	3.39	2.04	11.11	0.02	0.03	0.03	0.09
65	PXN	39.08	40.69	28.57	33.33	0.84	0.72	0.53	1.53
66	PLEC	64.97	40.47	1.02	55.56	1.15	0.56	0	1.03
67	TLN1	88.74	44.07	61.22	66.67	2.13	0.54	1.05	1.49
68	ACTG1	99.04	99.8	100	100	7.29	7.84	8.25	8.01
69	IGF2BP1	0.19	0.69	0	0	0	0.01	0	0
70	VASP	32.15	4.26	2.04	0	0.52	0.05	0.03	0
71	NF2	88.64	97.33	87.76	77.78	1.5	2.79	1.6	1.09
72	PAK2	62.85	79.91	73.47	55.56	1.25	1.69	1.46	1.21
73	STK3	26.28	18.81	41.84	11.11	0.67	0.46	1.11	0.21
74	HSPG2	3.56	7.44	4.08	0	0.02	0.08	0.05	0
75	NCK1	30.22	36.2	16.33	0	1.14	1.23	0.49	0
76	PAK1	38.5	3.63	21.43	11.11	1.08	0.07	0.48	0.41

77	DNM1L	49.66	80.67	70.41	22.22	0.78	1.88	1.63	0.41
78	ACTB	100	100	100	100	9.61	7.97	9.22	9.18
79	EZR	84.79	32.25	7.14	77.78	3.52	1.02	0.17	3.98
80	ROCK2	23.29	46.93	31.63	0	0.21	0.61	0.17	0
81	USP9X	87.97	92.92	83.67	77.78	1.93	2.02	1.94	2.28
82	MYH14	1.15	15.09	30.61	0	0	0.13	0.42	0
83	CCDC141	2.21	3.85	2.04	0	0	0.02	0	0
84	CLIP1	33.49	32.53	24.49	11.11	0.63	0.6	0.5	0.12
85	CDC42	95.28	94.98	94.9	77.78	3.91	3.77	4.21	3.73
86	ITGA5	37.92	1.79	1.02	11.11	1.03	0.03	0	0.06
87	DNM2	53.32	33.27	63.27	11.11	1.05	0.57	1.46	0.46
88	TOR1A	40.9	53.64	38.78	0	1.37	1.77	1.21	0
89	ACTC1	2.31	8.57	0	0	0.01	0.15	0	0
90	CORO1A	90.66	9.89	3.06	100	5.17	0.32	0.08	6.71
91	DCTN1	54.38	88.21	96.94	55.56	1.45	3.02	4.37	1.4
92	PFN1	97.21	93.49	91.84	100	4.94	3.73	3.79	6.39
93	ACTR3	80.56	70.3	57.14	66.67	3.51	2.54	2.16	2.34
94	DIAPH1	13.86	35.49	2.04	55.56	0.21	0.62	0.01	1.84
95	RANBP1	35.42	65.33	44.9	22.22	1.26	2.52	1.65	0.98

96	KIF3A	70.16	92.84	86.73	33.33	0.39	2.02	1.56	0.18
97	KIF3B	23.29	55.29	32.65	22.22	0.21	0.9	0.34	0.64
98	PRKG1	0.87	0.88	1.02	0	0.01	0.01	0.01	0
99	IQGAP1	32.44	13.79	80.61	55.56	0.59	0.16	1.7	1.83

Supplementary Table 6. Analysis of gene expression in the scRNAseq GBM dataset

#	id/CLUSTER	Fraction Expressing Gene X				Scaled Mean Expression of Gene X			
		**microglia/macrophage**	**malignant**	**oligodendrocytes**	**T cells**	**microglia/macrophage**	**malignant**	**oligodendrocytes**	**T cells**
1	PALLD	24.67	62.17	4.57	1.06	0.56	1.36	0.07	0.01
2	MYLK	1.86	9.72	73.97	2.13	0.04	0.15	3.56	0.03
3	TTN	30.9	44.11	22.83	45.74	0.1	0.13	0.05	0.51
4	TPM1	15.92	42.07	5.94	3.19	0.44	1.38	0.14	0.08
5	VCL	31.03	39.11	8.22	18.09	0.7	0.85	0.13	0.31
6	FERMT2	1.33	43.7	37.44	2.13	0.02	1.34	1.06	0.05
7	ITGB1	75.07	62.23	60.73	58.51	2.94	2.04	2.15	2.38
8	LPP	99.47	99.66	100	100	1.42	1.89	1.23	1.4
9	CALD1	3.05	78.25	63.01	1.06	0.05	2.3	1.53	0
10	PARVA	21.62	56.55	11.87	19.15	0.03	0.92	0.03	0.02
11	LIMA1	9.28	80.45	33.79	10.64	0.21	3.35	1.03	0.32

12	CTNND1	58.49	55.98	39.73	2.13	0.57	0.56	0.38	0.01
13	MYPN	0.13	0.22	0	0	0	0	0	0
14	ITGAV	63.66	66.2	47.95	8.51	1.49	1.42	0.98	0.06
15	MYL9	1.99	5.41	1.37	1.06	0.04	0.1	0.02	0.02
16	ACTA2	9.42	8.76	0.46	14.89	0.26	0.28	0.02	0.49
17	ENAH	86.6	97.74	92.24	91.49	0.19	1.98	1.31	0.24
18	DCC	0.66	32.7	5.48	1.06	0	0.67	0.08	0
19	OBSCN	4.77	17.56	0.46	23.4	0.02	0.1	0	0.21
20	CAV1	0.8	12.82	23.74	2.13	0.02	0.46	0.81	0.04
21	CD151	31.56	68.31	6.85	15.96	0.62	1.77	0.1	0.3
22	CAV2	43.63	56.78	52.97	52.13	0.15	0.54	0.96	0.21
23	MYOT	3.05	6	21.92	0	0.04	0.14	0.68	0
24	HMCN1	0.4	1.85	0.46	1.06	0	0.01	0	0
25	CNN1	0.13	1.25	0.46	0	0	0.03	0	0
26	RHOC	49.34	70.71	19.18	26.6	1.98	3.31	0.67	1.33
27	FLNB	13.79	23.28	44.75	11.7	0.18	0.33	0.72	0.19
28	LMNA	39.92	65.09	86.76	22.34	1.33	2.28	3.8	0.72
29	MUSK	0.66	1.27	8.22	0	0	0.01	0.14	0
30	CXADR	70.03	83.74	90.41	70.21	1.06	2.25	3.16	0.28

31	FSCN1	61.67	86.03	97.26	5.32	1.36	2.46	3.4	0.07
32	SPEG	0.27	28.69	0.46	3.19	0	0.49	0.01	0.1
33	ITGB5	20.03	6.62	6.85	0	0.42	0.13	0.16	0
34	ALPK3	24.4	9.72	0	3.19	0.35	0.1	0	0.02
35	CFL1	98.67	99.04	99.54	97.87	6.02	6.26	6.24	6.41
36	TJP1	5.84	68.42	89.95	2.13	0.06	1.29	2.46	0.03
37	RAC1	80.9	85.76	80.82	26.6	2.6	2.89	2.52	0.58
38	ACTG2	2.92	3.09	1.37	1.06	0.02	0.03	0.01	0
39	IGFN1	0.27	0.35	0	0	0	0	0	0
40	WASL	24.54	73.39	52.97	9.57	0.38	1.86	1.2	0.21
41	FLNA	31.3	79.6	12.79	70.21	0.68	1.94	0.18	1.93
42	ACTN4	34.62	76.38	87.21	55.32	0.73	2.17	3.06	1.71
43	PDGFRA	1.33	53.94	4.57	0	0	2.3	0.01	0
44	MYH9	87.8	48.65	59.82	95.74	2.17	0.81	1.17	3.74
45	GSN	87.8	30.19	99.09	13.83	5.26	1.2	7.76	0.42
46	CTTN	1.72	90.62	94.98	3.19	0.04	3.26	4.05	0.06
47	DBN1	1.86	76.98	2.74	1.06	0.02	3.14	0.02	0.03
48	MYO6	37.93	74.33	93.15	29.79	0.22	1.63	3.51	0.16
49	AMOT	7.56	15.65	4.57	1.06	0.13	0.3	0.05	0.01

50	JUP	8.22	9.69	40.18	0	0.22	0.23	1.1	0
51	MYH10	13.13	68.38	9.59	5.32	0.11	1.66	0.1	0.03
52	PAFAH1B1	50.27	69.04	59.82	26.6	0.83	1.61	1.39	0.63
53	PTPN12	34.22	54.17	25.11	5.32	0.88	1.46	0.69	0.16
54	PDGFRB	0.93	9.75	0.91	1.06	0.02	0.24	0.02	0.05
55	RHOA	96.15	92.77	99.54	81.91	5.77	5.11	6.09	4.88
56	SPTAN1	36.74	84.19	22.83	51.06	0.52	2.14	0.42	1.34
57	ACTN1	22.55	35.67	5.02	2.13	0.65	1.07	0.07	0.04
58	FLNC	0.4	20.62	32.42	1.06	0	0.5	0.66	0
59	RRAS	7.43	3.95	1.37	0	0.22	0.11	0.05	0
60	MARK2	24.01	26.29	30.59	22.34	0.19	0.23	0.3	0.27
61	FLII	68.7	53.82	55.25	37.23	2.16	1.41	1.45	1.17
62	ARHGEF7	50.93	77.58	79.45	45.74	1.09	2.3	2.3	1.13
63	ARHGAP35	26.92	66.2	37.9	32.98	0.29	1.15	0.56	0.49
64	ACTA1	2.39	4.07	1.83	2.13	0.02	0.04	0	0.01
65	PXN	38.99	48.1	24.2	30.85	0.73	0.98	0.39	1.06
66	PLEC	62.73	47.11	3.2	52.13	1.13	0.69	0.04	1.16
67	TLN1	83.16	47.69	57.99	60.64	1.74	0.66	0.97	1.33
68	ACTG1	94.83	99.45	99.54	95.74	6.26	8.12	8	6.77

69	IGF2BP1	0.13	6.21	0.91	1.06	0	0.09	0	0
70	VASP	22.55	6.25	2.74	6.38	0.35	0.08	0.04	0.15
71	NF2	94.43	97.04	90.41	97.87	1.27	2.19	1.42	1.61
72	PAK2	66.58	77.94	83.56	52.13	1.27	1.72	1.89	1.26
73	STK3	24.67	28.6	36.99	2.13	0.65	0.74	1.07	0.06
74	HSPG2	2.52	14.43	7.31	2.13	0.02	0.13	0.07	0
75	NCK1	29.18	33.16	11.87	21.28	1.17	1.18	0.39	0.99
76	PAK1	47.21	18.21	14.16	19.15	1.41	0.42	0.38	0.53
77	DNM1L	56.5	77.71	68.49	48.94	0.74	1.82	1.3	0.79
78	ACTB	100	99.9	100	100	9.26	8.89	9.26	9.16
79	EZR	60.88	54.13	4.57	82.98	2.22	1.97	0.12	3.94
80	ROCK2	23.61	50.76	28.31	17.02	0.2	0.66	0.39	0.2
81	USP9X	87.67	91.99	83.11	77.66	1.77	2.16	1.67	1.33
82	MYH14	0.4	8.66	28.77	0	0	0.08	0.37	0
83	CCDC141	4.77	4.28	2.74	10.64	0.01	0.03	0	0.2
84	CLIP1	32.49	45.59	24.2	21.28	0.63	0.91	0.49	0.51
85	CDC42	93.1	93.85	94.98	76.6	3.7	3.87	3.87	3.09
86	ITGA5	50.66	17.32	1.83	20.21	1.52	0.47	0.05	0.48
87	DNM2	55.97	32.57	53.42	24.47	1.15	0.57	1.25	0.84

88	TOR1A	39.26	48.65	26.94	20.21	1.42	1.67	0.79	0.71
89	ACTC1	2.25	5.35	0.91	1.06	0	0.09	0	0
90	CORO1A	84.35	11.26	0.46	88.3	4.72	0.37	0	5.8
91	DCTN1	46.95	79.62	93.61	22.34	1.16	2.55	4.01	0.72
92	PFN1	95.89	91.26	95.43	100	4.97	3.92	4.34	5.97
93	ACTR3	76.79	74.75	57.08	65.96	3.67	3.21	2.53	3.28
94	DIAPH1	20.56	39.75	3.65	47.87	0.34	0.74	0.08	1.18
95	RANBP1	27.85	57.35	36.07	17.02	1.06	2.32	1.37	0.75
96	KIF3A	80.9	92.1	85.39	81.91	0.4	1.76	1.59	0.63
97	KIF3B	34.35	58.81	28.77	30.85	0.23	0.94	0.33	0.35
98	PRKG1	0.27	1.81	0.46	0	0	0.02	0.01	0
99	IQGAP1	72.28	48.16	79	76.6	1.8	0.95	1.69	2.53
