# Supplementary material for: Cytoskeletal Protein Palladin in Adult Gliomas Predicts Disease Incidence, Progression, and Prognosis
Source: Cancers (Basel). 2022 Oct 19;14(20):5130. doi: 10.3390/cancers14205130 (PMC9600953; doi:10.3390/cancers14205130)
Supplement: Supplementary file 1 [file cancers-14-05130-s001.zip › supp_table_3.pdf]

Name	CNS2081
Description	Central nerve disease spectrum (central nerve tumor progression) tissue array, 104 cases/208 cores
Panel	Central nerve tumor tissue microarray, containing 24 cases of astrocytoma, 8 each of glioblastoma, ol
Price	474
Cases	104
Cores	208
Diameter	5
Rows	13
Columns	16

Position	No.	Age	Sex	Organ/Anatomic Site	Pathology diagnosis
A1	1	62	M	Cerebrum	Astrocytoma
A2	2	62	M	Cerebrum	Astrocytoma
A3	3	31	M	Cerebrum	Astrocytoma
A4	4	31	M	Cerebrum	Astrocytoma
A5	5	52	M	Cerebrum	Astrocytoma
A6	6	52	M	Cerebrum	Astrocytoma
A7	7	41	M	Cerebrum	Astrocytoma
A8	8	41	M	Cerebrum	Astrocytoma
A9	9	36	F	Cerebrum	Astrocytoma (brain tissue)
A10	10	36	F	Cerebrum	Astrocytoma
A11	11	33	M	Cerebrum	Astrocytoma
A12	12	33	M	Cerebrum	Astrocytoma
A13	13	29	M	Cerebrum	Astrocytoma
A14	14	29	M	Cerebrum	Astrocytoma
A15	15	46	F	Cerebrum	Astrocytoma
A16	16	46	F	Cerebrum	Astrocytoma
B1	17	17	M	Cerebrum	Astrocytoma
B2	18	17	M	Cerebrum	Astrocytoma
B3	19	10	M	Cerebellum	Astrocytoma
B4	20	10	M	Cerebellum	Astrocytoma
B5	21	46	M	Cerebrum	Astrocytoma
B6	22	46	M	Cerebrum	Astrocytoma
B7	23	16	F	Cerebrum	Astrocytoma
B8	24	16	F	Cerebrum	Astrocytoma
B9	25	36	F	Cerebrum	Astrocytoma
B10	26	36	F	Cerebrum	Astrocytoma
B11	27	53	M	Cerebrum	Astrocytoma
B12	28	53	M	Cerebrum	Astrocytoma
B13	29	40	M	Cerebrum	Astrocytoma
B14	30	40	M	Cerebrum	Astrocytoma
B15	31	50	F	Cerebrum	Astrocytoma
B16	32	50	F	Cerebrum	Astrocytoma
C1	33	48	M	Cerebrum	Astrocytoma
C2	34	48	M	Cerebrum	Astrocytoma
C3	35	36	F	Cerebrum	Astrocytoma
C4	36	36	F	Cerebrum	Astrocytoma
C5	37	36	F	Cerebrum	Astrocytoma
C6	38	36	F	Cerebrum	Astrocytoma
C7	39	61	M	Cerebrum	Astrocytoma
C8	40	61	M	Cerebrum	Astrocytoma
C9	41	40	M	Cerebrum	Astrocytoma
C10	42	40	M	Cerebrum	Astrocytoma

C11	43	41	F	Cerebrum	Astrocytoma
C12	44	41	F	Cerebrum	Astrocytoma
C13	45	52	F	Cerebrum	Astrocytoma
C14	46	52	F	Cerebrum	Astrocytoma
C15	47	56	M	Cerebrum	Astrocytoma
C16	48	56	M	Cerebrum	Astrocytoma
D1	49	31	M	Cerebrum	Glioblastoma
D2	50	31	M	Cerebrum	Glioblastoma
D3	51	42	M	Cerebrum	Glioblastoma
D4	52	42	M	Cerebrum	Glioblastoma
D5	53	68	F	Cerebellum	Glioblastoma (cerebellar tissue)
D6	54	68	F	Cerebellum	Glioblastoma (sparse)
D7	55	65	F	Cerebrum	Glioblastoma
D8	56	65	F	Cerebrum	Glioblastoma
D9	57	9	M	Cerebrum	Glioblastoma
D10	58	9	M	Cerebrum	Glioblastoma
D11	59	43	M	Cerebrum	Glioblastoma
D12	60	43	M	Cerebrum	Glioblastoma
D13	61	33	F	Cerebrum	Glioblastoma
D14	62	33	F	Cerebrum	Glioblastoma
D15	63	37	M	Cerebrum	Glioblastoma
D16	64	37	M	Cerebrum	Glioblastoma
E1	65	52	M	Cerebrum	Oligodendroglioma
E2	66	52	M	Cerebrum	Oligodendroglioma
E3	67	56	F	Cerebrum	Oligodendroglioma
E4	68	56	F	Cerebrum	Oligodendroglioma
E5	69	38	M	Cerebellum	Oligodendroglioma
E6	70	38	M	Cerebellum	Oligodendroglioma
E7	71	30	F	Cerebrum	Oligodendroglioma
E8	72	30	F	Cerebrum	Oligodendroglioma
E9	73	39	M	Cerebrum	Oligodendroglioma
E10	74	39	M	Cerebrum	Oligodendroglioma
E11	75	17	M	Cerebrum	Malignant oligodendroglioma
E12	76	17	M	Cerebrum	Malignant oligodendroglioma
E13	77	43	M	Cerebrum	Malignant oligodendroglioma
E14	78	43	M	Cerebrum	Malignant oligodendroglioma
E15	79	73	M	Cerebrum	Malignant oligodendroglioma
E16	80	73	M	Cerebrum	Malignant oligodendroglioma
F1	81	41	F	Cerebrum	Ependymoma
F2	82	41	F	Cerebrum	Ependymoma
F3	83	46	M	Cerebrum	Ependymoma
F4	84	46	M	Cerebrum	Ependymoma
F5	85	55	F	Cerebrum	Ependymoma
F6	86	55	F	Cerebrum	Ependymoma
F7	87	5	M	Cerebellum	Malignant ependymoma
F8	88	5	M	Cerebellum	Malignant ependymoma
F9	89	33	M	Cerebrum	Malignant ependymoma
F10	90	33	M	Cerebrum	Malignant ependymoma
F11	91	24	F	Cerebrum	Malignant ependymoma
F12	92	24	F	Cerebrum	Malignant ependymoma
F13	93	18	M	Cerebrum	Malignant ependymoma
F14	94	18	M	Cerebrum	Malignant ependymoma
F15	95	29	F	Cerebrum	Malignant ependymoma

F16	96	29	F	Cerebrum	Malignant ependymoma
G1	97	3	F	Cerebrum	Medulloblastoma
G2	98	3	F	Cerebrum	Medulloblastoma
G3	99	11	M	Cerebrum	Medulloblastoma
G4	100	11	M	Cerebrum	Medulloblastoma
G5	101	47	F	Cerebellum	Medulloblastoma
G6	102	47	F	Cerebellum	Medulloblastoma
G7	103	30	M	Cerebellum	Medulloblastoma
G8	104	30	M	Cerebellum	Medulloblastoma
G9	105	8	F	Cerebellum	Medulloblastoma
G10	106	8	F	Cerebellum	Medulloblastoma
G11	107	32	F	Cerebellum	Medulloblastoma
G12	108	32	F	Cerebellum	Medulloblastoma
G13	109	14	F	Cerebrum	Medulloblastoma
G14	110	14	F	Cerebrum	Medulloblastoma
G15	111	33	F	Cerebellum	Medulloblastoma
G16	112	33	F	Cerebellum	Medulloblastoma
H1	113	14	M	Cerebrum	Medulloblastoma
H2	114	14	M	Cerebrum	Medulloblastoma
H3	115	7	M	Cerebellum	Medulloblastoma
H4	116	7	M	Cerebellum	Medulloblastoma
H5	117	50	M	Cerebellum	Malignant meningioma
H6	118	50	M	Cerebellum	Malignant meningioma
H7	119	43	M	Cerebrum	Malignant meningioma
H8	120	43	M	Cerebrum	Malignant meningioma
H9	121	63	F	Cerebrum	Meningothelial meningioma
H10	122	63	F	Cerebrum	Meningothelial meningioma (sparse)
H11	123	67	M	Cerebrum	Meningothelial meningioma
H12	124	67	M	Cerebrum	Meningothelial meningioma
H13	125	40	F	Cerebrum	Fibrous meningioma
H14	126	40	F	Cerebrum	Fibrous meningioma
H15	127	44	M	Cerebrum	Mixed meningioma
H16	128	44	M	Cerebrum	Mixed meningioma
I1	129	46	M	Cerebrum	Meningothelial meningioma
I2	130	46	M	Cerebrum	Meningothelial meningioma
I3	131	28	M	Cerebrum	Mixed meningioma
I4	132	28	M	Cerebrum	Mixed meningioma
I5	133	44	F	Cerebrum	Mixed meningioma
I6	134	44	F	Cerebrum	Mixed meningioma
I7	135	61	F	Cerebrum	Mixed meningioma
I8	136	61	F	Cerebrum	Mixed meningioma
I9	137	47	F	Cerebrum	Mixed meningioma
I10	138	47	F	Cerebrum	Mixed meningioma
I11	139	29	F	Cerebrum	Mixed meningioma
I12	140	29	F	Cerebrum	Mixed meningioma
I13	141	74	M	Cerebrum	Mixed meningioma
I14	142	74	M	Cerebrum	Mixed meningioma
I15	143	38	M	Cerebrum	Mixed meningioma
I16	144	38	M	Cerebrum	Mixed meningioma
J1	145	25	F	Cerebellum	Mixed meningioma
J2	146	25	F	Cerebellum	Mixed meningioma
J3	147	38	M	Cerebrum	Fibrous meningioma
J4	148	38	M	Cerebrum	Fibrous meningioma

J5	149	35	F	Cerebrum	Fibrous meningioma
J6	150	35	F	Cerebrum	Fibrous meningioma
J7	151	36	F	Cerebrum	Fibrous meningioma
J8	152	36	F	Cerebrum	Fibrous meningioma
J9	153	46	F	Cerebrum	Fibrous meningioma
J10	154	46	F	Cerebrum	Fibrous meningioma
J11	155	31	F	Cerebellum	Fibrous meningioma
J12	156	31	F	Cerebellum	Fibrous meningioma
J13	157	37	F	Cerebrum	Fibrous meningioma
J14	158	37	F	Cerebrum	Fibrous meningioma
J15	159	60	F	Cerebrum	Fibrous meningioma
J16	160	60	F	Cerebrum	Fibrous meningioma
K1	161	48	F	Cerebrum	Fibrous meningioma
K2	162	48	F	Cerebrum	Fibrous meningioma
K3	163	38	F	Cerebrum	Microcystic meningioma
K4	164	38	F	Cerebrum	Microcystic meningioma
K5	165	32	M	Cerebrum	Hyperplasia of gliocyte
K6	166	32	M	Cerebrum	Hyperplasia of gliocyte
K7	167	30	M	Cerebrum	Hyperplasia of gliocyte
K8	168	30	M	Cerebrum	Hyperplasia of gliocyte
K9	169	31	M	Cerebrum	Chronic abscess
K10	170	31	M	Cerebrum	Chronic abscess
K11	171	18	F	Cerebrum	Tuberculous inflammation
K12	172	18	F	Cerebrum	Tuberculous inflammation (cheesy necrosis)
K13	173	40	F	Cerebrum	Chronic inflammation
K14	174	40	F	Cerebrum	Chronic inflammation
K15	175	34	F	Cerebrum	Chronic inflammation
K16	176	34	F	Cerebrum	Chronic inflammation
L1	177	37	F	Cerebrum	Cancer adjacent normal cerebral tissue
L2	178	37	F	Cerebrum	Cancer adjacent normal cerebral tissue
L3	179	39	M	Cerebrum	Cancer adjacent normal cerebral tissue
L4	180	39	M	Cerebrum	Cancer adjacent normal cerebral tissue
L5	181	47	F	Cerebrum	Cancer adjacent normal cerebral tissue
L6	182	47	F	Cerebrum	Cancer adjacent normal cerebral tissue
L7	183	38	M	Cerebrum	Cancer adjacent normal cerebral tissue
L8	184	38	M	Cerebrum	Cancer adjacent normal cerebral tissue
L9	185	30	M	Cerebrum	Cancer adjacent normal cerebral tissue
L10	186	30	M	Cerebrum	Cancer adjacent normal cerebral tissue
L11	187	38	M	Cerebrum	Cancer adjacent normal medulla oblongata tissue
L12	188	38	M	Cerebrum	Cancer adjacent normal medulla oblongata tissue
L13	189	38	F	Cerebrum	Cancer adjacent normal cerebral tissue
L14	190	38	F	Cerebrum	Cancer adjacent normal cerebral tissue
L15	191	49	F	Cerebrum	Cancer adjacent normal cerebral tissue
L16	192	49	F	Cerebrum	Cancer adjacent normal cerebral tissue
M1	193	2	F	Cerebrum	Normal cerebral tissue
M2	194	2	F	Cerebrum	Normal cerebral tissue
M3	195	50	F	Cerebrum	Normal cerebral tissue
M4	196	50	F	Cerebrum	Normal cerebral tissue
M5	197	38	F	Cerebrum	Normal cerebral tissue
M6	198	38	F	Cerebrum	Normal cerebral tissue (sparse)
M7	199	28	F	Cerebellum	Normal cerebellar tissue
M8	200	28	F	Cerebellum	Normal cerebellar tissue
M9	201	58	F	Cerebellum	Normal cerebellar tissue

M10	202	58	F	Cerebellum	Normal cerebellar tissue
M11	203	8	F	Cerebellum	Normal cerebellar tissue
M12	204	8	F	Cerebellum	Normal cerebellar tissue
M13	205	24	F	Cerebellum	Normal cerebellar tissue
M14	206	24	F	Cerebellum	Normal cerebellar tissue
M15	207	26	M	Cerebellum	Normal cerebellar tissue
M16	208	26	M	Cerebellum	Normal cerebellar tissue

igodendroglioma and ependymoma, 10 medulloblastoma, 24 meningioma, 2 hyperplasia, 4 inflammation,

TNM	Grade	Stage	Type	Tissue ID.	% Nuceli
	1		malignant	Nct030267	40
	1		malignant	Nct030267	60
	1		malignant	Nct030292	30
	1		malignant	Nct030292	30
	1		malignant	Nct030106	30
	1		malignant	Nct030106	70
	1		malignant	Nct030068	50
	1		malignant	Nct030068	5
	-		malignant	Nct030041	10
	1		malignant	Nct030041	1
	2		malignant	Nct030286	1
	1		malignant	Nct030286	3
	2		malignant	Nct020029	3
	2		malignant	Nct020029	20
	2		malignant	Nct030023	0
	2		malignant	Nct030023	0
	2		malignant	Nct010092	1
	2		malignant	Nct010092	2
	2		malignant	Ncb040034	2
	2		malignant	Ncb040034	2
	2		malignant	Nct010014	0
	2		malignant	Nct010014	5
	2		malignant	Nct030109	3
	2		malignant	Nct030109	0
	2		malignant	Nct030153	0
	2		malignant	Nct030153	10
	2		malignant	Nct030043	5
	2		malignant	Nct030043	5
	2		malignant	Nct030026	NA
	2		malignant	Nct030026	20
	2		malignant	Nct030359	0
	2		malignant	Nct030359	0
	2--3		malignant	Nct030259	0
	2--3		malignant	Nct030259	30
	3		malignant	Nct030039	0
	3		malignant	Nct030039	0
	2		malignant	Nct030049	0
	2		malignant	Nct030049	0
	3		malignant	Nct030074	0
	3		malignant	Nct030074	0
	2--3		malignant	Nct030107	0
	3		malignant	Nct030107	0

2--3	malignant	Nct030025	0
2--3	malignant	Nct030025	0
3	malignant	Nct030256	20
3	malignant	Nct030256	10
3	malignant	Nct030284	0
3	malignant	Nct030284	0
-	malignant	Nct020084	20
-	malignant	Nct020084	0
-	malignant	Nct020002	0
-	malignant	Nct020002	0
-	malignant	Ncb060172	0
-	malignant	Ncb060172	0
-	malignant	Nct010115	20
-	malignant	Nct010115	20
-	malignant	Nct020175	5
-	malignant	Nct020175	20
-	malignant	Nct030339	0
-	malignant	Nct030339	20
-	malignant	Nct050240	1
-	malignant	Nct050240	0
-	malignant	Nct050164	0
-	malignant	Nct050164	0
-	malignant	Nct020087	5
-	malignant	Nct020087	0
-	malignant	Nct030191	0
-	malignant	Nct030191	0
-	malignant	Ncb040308	0
-	malignant	Ncb040308	0
-	malignant	Nct030173	40
-	malignant	Nct030173	20
-	malignant	Nct030029	5
-	malignant	Nct030029	0
-	malignant	Nct020280	NA
-	malignant	Nct020280	60
-	malignant	Nct010117	0
-	malignant	Nct010117	0
-	malignant	Nct030229	0
-	malignant	Nct030229	0
-	malignant	Nct040299	40
-	malignant	Nct040299	40
-	malignant	Nct060144	0
-	malignant	Nct060144	0
-	malignant	Nct040008	3
-	malignant	Nct040008	0
-	malignant	Ncb040108	0
-	malignant	Ncb040108	0
-	malignant	Nct020057	0
-	malignant	Nct020057	40
-	malignant	Nct060222	NA
-	malignant	Nct060222	0
-	malignant	Nct020093	3
-	malignant	Nct020093	3
-	malignant	Nct030157	20

-	malignant	Nct030157	20
-	malignant	Nct020235	20
-	malignant	Nct020235	20
-	malignant	Nct020003	NA
-	malignant	Nct020003	NA
-	malignant	Ncb040185	0
-	malignant	Ncb040185	1
-	malignant	Ncb040371	0
-	malignant	Ncb040371	0
-	malignant	Ncb050014	0
-	malignant	Ncb050014	0
-	malignant	Ncb050040	0
-	malignant	Ncb050040	0
-	malignant	Nct010023	0
-	malignant	Nct010023	0
-	malignant	Ncb050121	0
-	malignant	Ncb050121	0
-	malignant	Nct020239	0
-	malignant	Nct020239	0
-	malignant	Ncb060010	0
-	malignant	Ncb060010	0
-	malignant	Ncb060258	0
-	malignant	Ncb060258	0
-	malignant	Nct010105	40
-	malignant	Nct010105	40
-	benign	Nct010110	30
-	benign	Nct010110	20
-	benign	Nct010078	10
-	benign	Nct010078	10
-	benign	Nct010037	30
-	benign	Nct010037	30
-	benign	Nct010054	30
-	benign	Nct010054	30
-	benign	Nct010056	0
-	benign	Nct010056	0
-	benign	Nct020001	50
-	benign	Nct020001	NA
-	benign	Nct010052	30
-	benign	Nct010052	30
-	benign	Nct010042	NA
-	benign	Nct010042	0
-	benign	Nct010004	80
-	benign	Nct010004	20
-	benign	Nct010002	0
-	benign	Nct010002	40
-	benign	Nct020007	5
-	benign	Nct020007	0
-	benign	Nct010060	40
-	benign	Nct010060	40
-	benign	Ncb050068	5
-	benign	Ncb050068	30
-	benign	Nct010087	30
-	benign	Nct010087	30

-	benign	Nct010005	10
-	benign	Nct010005	20
-	benign	Nct010024	3
-	benign	Nct010024	20
-	benign	Nct020018	70
-	benign	Nct020018	80
-	benign	Ncb050177	50
-	benign	Ncb050177	0
-	benign	Nct020006	70
-	benign	Nct020006	70
-	benign	Nct010040	0
-	benign	Nct010040	0
-	benign	Nct020016	0
-	benign	Nct020016	0
-	benign	Nct010091	20
-	benign	Nct010091	20
-	hyperplasia	Nct020292	70
-	hyperplasia	Nct020292	70
-	hyperplasia	Nct020088	80
-	hyperplasia	Nct020088	80
-	inflammation	Nct030358	90
-	inflammation	Nct030358	90
-	inflammation	Nct030060	0
-	inflammation	Nct030060	0
-	inflammation	Nct030231	0
-	inflammation	Nct030231	0
-	inflammation	Nct030218	0
-	inflammation	Nct030218	NA
-	NAT	Nct020226	0
-	NAT	Nct020226	2
-	NAT	Nct020245	0
-	NAT	Nct020245	0
-	NAT	Nct030126	0
-	NAT	Nct030126	0
-	NAT	Nct030198	0
-	NAT	Nct030198	0
-	NAT	Nct030249	0
-	NAT	Nct030249	0
-	NAT	Nct030276	20
-	NAT	Nct030276	0
-	NAT	Nct030303	0
-	NAT	Nct030303	0
-	NAT	Nct030366	20
-	NAT	Nct030366	0
-	normal	Nct03N002	0
-	normal	Nct03N002	0
-	normal	Nct03N005	0
-	normal	Nct03N005	0
-	normal	Nct03N008	0
-	normal	Nct03N008	0
-	normal	Ncb03N013	0
-	normal	Ncb03N013	0
-	normal	Ncb04N008	0

-	normal	Ncb04N008	0
-	normal	Ncb04N012	0
-	normal	Ncb04N012	0
-	normal	Ncb04N001	0
-	normal	Ncb04N001	0
-	normal	Ncb05N008	0
-	normal	Ncb05N008	0

, 8 each of adjacent normal tissue and normal tissue, duplicate cores per case

Cancer cells			
Nuclei intensity	**%** Membrane	Membrane intensity	**%** Cytoplasme
8	60	5	0
8	70	5	0
8	80	7	0
9	70	7	0
7	40	5	0
6	70	6	0
4	20	4	0
3	30	3	10
3	90	5	0
4	2	3	0
4	90	3	0
4	90	3	0
3	80	6	0
3	60	4	0
0	95	7	0
0	95	7	0
5	100	5	0
6	100	5	0
5	30	5	100
5	30	5	100
0	80	7	0
5	100	4	0
5	100	4	0
0	100	7	30
0	100	6	30
5	100	4	50
6	100	4	20
1	80	3	0
NA	NA	NA	NA
4	30	3	20
0	100	4	20
0	100	3	100
0	80	4	0
3	100	3	80
0	80	3	100
0	100	4	80
0	100	6	20
0	100	6	20
0	100	4	20
0	100	3	20
0	100	2	0
0	100	2	0

0	40	3	30
0	30	2	80
4	100	3	0
6	100	5	0
0	100	4	0
0	100	4	0
4	100	4	0
0	70	2	0
0	80	3	50
0	100	4	10
0	100	6	100
0	100	6	100
5	100	3	100
6	100	2	100
3	100	2	100
3	100	3	20
0	90	3	90
2	80	2	0
3	100	3	100
0	80	2	80
0	100	4	0
0	90	3	30
3	100	3	100
0	100	4	80
0	100	5	80
0	100	5	80
0	0	0	60
0	0	0	60
2	0	0	50
1	100	1	100
2	100	3	10
0	100	1	100
NA	NA	NA	NA
2	95	2	5
0	90	3	90
0	90	3	90
0	80	1	80
0	80	2	80
7	100	2	100
7	100	2	100
0	100	6	100
0	100	6	100
5	100	3	100
0	100	4	0
0	100	6	100
0	90	6	90
0	15	4	0
2	70	4	70
NA	NA	NA	NA
0	100	4	100
4	100	4	100
4	100	4	100
4	0	0	60

4	0	0	60
4	100	3	100
4	100	3	100
NA	NA	NA	NA
NA	NA	NA	NA
0	80	3	0
5	80	3	80
0	100	2	0
0	100	2	0
0	100	2	100
0	100	2	100
0	60	1	0
0	60	1	0
0	0	0	0
0	0	0	0
0	70	1	0
0	70	1	0
0	0	0	0
0	0	0	0
0	0	0	70
0	0	0	70
0	0	0	80
0	0	0	0
4	0	0	100
4	0	0	100
4	100	2	0
5	100	2	0
3	100	2	100
4	100	2	100
4	0	0	100
4	100	2	100
4	100	2	100
4	100	2	100
0	100	2	100
0	100	2	100
3	100	2	100
NA	NA	NA	NA
4	100	2	100
4	100	2	100
NA	NA	NA	NA
0	100	2	100
2	100	2	100
2	100	2	100
0	100	1	100
2	100	2	100
2	100	2	100
0	100	2	100
3	100	2	100
3	100	2	100
3	100	2	100
3	100	2	100
4	100	2	100
4	100	2	100

3	100	2	100
2	100	2	100
2	100	2	100
1	100	1	100
4	100	2	100
5	100	2	100
2	100	2	100
0	100	2	100
5	100	3	100
5	100	3	100
0	100	1	100
0	100	1	100
0	100	2	100
0	100	2	100
2	100	3	100
2	100	3	100
2	0	0	0
2	0	0	0
4	0	0	0
4	0	0	0
4	80	4	0
4	80	4	0
0	20	2	0
0	20	2	0
0	0	0	2
0	0	0	50
0	30	3	0
NA	NA	NA	NA
0	0	0	0
2	0	0	0
0	0	0	0
0	0	0	0
0	0	0	20
0	0	0	30
0	0	0	40
0	0	0	10
0	0	0	20
0	0	0	40
2	0	0	0
0	0	0	20
0	0	0	20
0	0	0	20
1	0	0	0
0	0	0	0
0	0	0	0
0	0	0	0
0	0	0	0
0	0	0	30
0	0	0	0
0	0	0	0
0	90	3	0
0	0	0	0
0	0	0	0

0	0	0	0
0	0	0	0
0	0	0	0
0	0	0	0
0	0	0	0
0	0	0	0
0	0	0	0

				Endothelial Cells
Cytoplasm intensity	**%** Nuceli	Nuclei intensity	**%** Membrane	Memrane intensity
0	NA	NA	NA	NA
0	NA	NA	NA	NA
0	NA	NA	NA	NA
0	NA	NA	NA	NA
0	100	7	0	0
0	NA	NA	NA	NA
0	NA	NA	NA	NA
6	NA	NA	NA	NA
0	NA	NA	NA	NA
0	NA	NA	NA	NA
0	NA	NA	NA	NA
0	NA	NA	NA	NA
0	NA	NA	NA	NA
0	NA	NA	NA	NA
0	NA	NA	NA	NA
0	NA	NA	NA	NA
0	NA	NA	NA	NA
0	NA	NA	NA	NA
4	NA	NA	NA	NA
4	NA	NA	NA	NA
0	0	0	80	7
0	NA	NA	NA	NA
0	NA	NA	NA	NA
4	NA	NA	NA	NA
6	NA	NA	NA	NA
3	0	0	100	6
4	5	6	100	4
0	0	0	100	6
NA	NA	NA	NA	NA
4	NA	NA	NA	NA
3	NA	NA	NA	NA
2	NA	NA	NA	NA
0	NA	NA	NA	NA
3	NA	NA	NA	NA
3	NA	NA	NA	NA
3	0	0	100	5
7	NA	NA	NA	NA
7	NA	NA	NA	NA
3	0	0	100	4
3	0	0	0	0
0	NA	NA	NA	NA
0	0	0	100	5

3	0	0	0	0
2	NA	NA	NA	NA
0	NA	NA	NA	NA
0	NA	NA	NA	NA
0	0	0	100	3
0	0	0	100	3
0	0	0	100	5
0	NA	NA	NA	NA
4	NA	NA	NA	NA
5	NA	NA	NA	NA
6	0	0	100	7
6	0	0	100	6
3	NA	NA	NA	NA
2	NA	NA	NA	NA
2	NA	NA	NA	NA
6	NA	NA	NA	NA
2	NA	NA	NA	NA
0	NA	NA	NA	NA
3	5	?	0	0
2	10	?	0	0
0	NA	NA	NA	NA
4	NA	NA	NA	NA
3	NA	NA	NA	NA
3	NA	NA	NA	NA
7	NA	NA	NA	NA
7	NA	NA	NA	NA
2	NA	NA	NA	NA
2	NA	NA	NA	NA
2	NA	NA	NA	NA
1	NA	NA	NA	NA
4	NA	NA	NA	NA
1	NA	NA	NA	NA
NA	NA	NA	NA	NA
3	NA	NA	NA	NA
3	NA	NA	NA	NA
3	NA	NA	NA	NA
1	NA	NA	NA	NA
2	NA	NA	NA	NA
2	NA	NA	NA	NA
2	NA	NA	NA	NA
6	0	0	0	0
6	0	0	0	0
3	NA	NA	NA	NA
0	NA	NA	NA	NA
6	NA	NA	NA	NA
6	NA	NA	NA	NA
0	0	0	100	8
4	0	0	100	8
NA	NA	NA	NA	NA
4	NA	NA	NA	NA
4	NA	NA	NA	NA
4	NA	NA	NA	NA
2	NA	NA	NA	NA

2	NA	NA	NA	NA
3	NA	NA	NA	NA
3	NA	NA	NA	NA
NA	NA	NA	NA	NA
NA	NA	NA	NA	NA
0	NA	NA	NA	NA
3	NA	NA	NA	NA
0	NA	NA	NA	NA
0	NA	NA	NA	NA
2	NA	NA	NA	NA
2	NA	NA	NA	NA
0	NA	NA	NA	NA
0	NA	NA	NA	NA
0	0		100	7
0	NA	NA	NA	NA
0	NA	NA	NA	NA
0	NA	NA	NA	NA
0	0	0	0	0
0	NA	NA	NA	NA
1	0	0	0	0
1	0	0	0	0
1	0	0	0	0
0	0	0	0	0
1	40	6	0	0
1	40	6	0	0
0	0	0	0	0
0	0	0	0	0
2	20	6	0	0
2	0	0	0	0
2	NA	NA	NA	NA
2	NA	NA	NA	NA
2	NA	NA	NA	NA
2	NA	NA	NA	NA
2	NA	NA	NA	NA
2	NA	NA	NA	NA
2	NA	NA	NA	NA
NA	NA	NA	NA	NA
2	NA	NA	NA	NA
2	NA	NA	NA	NA
NA	NA	NA	NA	NA
2	NA	NA	NA	NA
2	NA	NA	NA	NA
2	NA	NA	NA	NA
1	NA	NA	NA	NA
2	NA	NA	NA	NA
2	NA	NA	NA	NA
2	0	0	0	0
2	NA	NA	NA	NA
2	NA	NA	NA	NA
2	NA	NA	NA	NA
2	NA	NA	NA	NA
2	NA	NA	NA	NA
2	NA	NA	NA	NA

2	0	0	0	0
2	0	0	0	0
2	NA	NA	NA	NA
1	NA	NA	NA	NA
2	NA	NA	NA	NA
2	0	0	0	0
2	NA	NA	NA	NA
2	NA	NA	NA	NA
3	NA	NA	NA	NA
3	NA	NA	NA	NA
1	NA	NA	NA	NA
1	NA	NA	NA	NA
2	NA	NA	NA	NA
2	NA	NA	NA	NA
3	NA	NA	NA	NA
3	NA	NA	NA	NA
0	0	0	100	4
0	0	0	0	0
0	NA	NA	NA	NA
0	NA	NA	NA	NA
0	0	0	0	0
0	0	0	0	0
0	NA	NA	NA	NA
0	NA	NA	NA	NA
2	0	0	0	0
4	0	0	0	0
0	0	0	40	3
NA	NA	NA	NA	NA
0	0	0	40	4
0	0	0	20	3
0	0	0	100	2
0	0	0	100	2
2	0	0	100	1
2	0	0	100	2
2	0	0	100	3
2	0	0	100	2
1	0	0	10	2
2	NA	NA	NA	NA
0	NA	NA	NA	NA
3	NA	NA	NA	NA
1	NA	NA	NA	NA
1	NA	NA	NA	NA
0	NA	NA	NA	NA
0	NA	NA	NA	NA
0	NA	NA	NA	NA
0	NA	NA	NA	NA
0	0	0	0	0
1	0	0	0	0
0	0	0	0	0
0	0	0	0	0
0	0	0	0	0
0	0	0	0	0
0	0	0	0	0

0	0	0	80	3
0	0	0	80	3
0	0	0	0	0
0	0	0	0	0
0	0	0	0	0
0	0	0	0	0
0	0	0	0	0

					**Lymphocytes**
**%** Cytoplasme	Cytoplasm intensity	**%** Nuceli	Nuclei intensity	**%** Membrane	Memrane intensity
NA	NA	NA	NA	NA	NA
NA	NA	NA	NA	NA	NA
NA	NA	NA	NA	NA	NA
NA	NA	NA	NA	NA	NA
100	7	NA	NA	NA	NA
NA	NA	NA	NA	NA	NA
NA	NA	NA	NA	NA	NA
NA	NA	NA	NA	NA	NA
NA	NA	NA	NA	NA	NA
NA	NA	NA	NA	NA	NA
NA	NA	NA	NA	NA	NA
NA	NA	NA	NA	NA	NA
NA	NA	NA	NA	NA	NA
NA	NA	NA	NA	NA	NA
NA	NA	NA	NA	NA	NA
NA	NA	NA	NA	NA	NA
NA	NA	NA	NA	NA	NA
NA	NA	NA	NA	NA	NA
NA	NA	NA	NA	NA	NA
NA	NA	NA	NA	NA	NA
0	0	NA	NA	NA	NA
NA	NA	NA	NA	NA	NA
NA	NA	NA	NA	NA	NA
NA	NA	NA	NA	NA	NA
NA	NA	NA	NA	NA	NA
0	0	NA	NA	NA	NA
0	0	NA	NA	NA	NA
0	0	NA	NA	NA	NA
NA	NA	NA	NA	NA	NA
NA	NA	NA	NA	NA	NA
NA	NA	NA	NA	NA	NA
NA	NA	NA	NA	NA	NA
NA	NA	NA	NA	NA	NA
NA	NA	NA	NA	NA	NA
NA	NA	NA	NA	NA	NA
100	5	0	0	0	0
NA	NA	NA	NA	NA	NA
NA	NA	NA	NA	NA	NA
100	4	NA	NA	NA	NA
0	0	NA	NA	NA	NA
NA	NA	NA	NA	NA	NA
0	0	NA	NA	NA	NA

100	3	NA	NA	NA	NA
NA	NA	NA	NA	NA	NA
NA	NA	NA	NA	NA	NA
NA	NA	NA	NA	NA	NA
100	3	NA	NA	NA	NA
100	3	NA	NA	NA	NA
100	5	NA	NA	NA	NA
NA	NA	NA	NA	NA	NA
NA	NA	NA	NA	NA	NA
NA	NA	NA	NA	NA	NA
100	7	NA	NA	NA	NA
100	6	NA	NA	NA	NA
NA	NA	NA	NA	NA	NA
NA	NA	NA	NA	NA	NA
NA	NA	NA	NA	NA	NA
NA	NA	NA	NA	NA	NA
NA	NA	NA	NA	NA	NA
NA	NA	NA	NA	NA	NA
0	0	NA	NA	NA	NA
0	0	NA	NA	NA	NA
NA	NA	NA	NA	NA	NA
NA	NA	NA	NA	NA	NA
NA	NA	NA	NA	NA	NA
NA	NA	NA	NA	NA	NA
NA	NA	NA	NA	NA	NA
NA	NA	NA	NA	NA	NA
NA	NA	NA	NA	NA	NA
NA	NA	NA	NA	NA	NA
NA	NA	NA	NA	NA	NA
NA	NA	NA	NA	NA	NA
NA	NA	NA	NA	NA	NA
NA	NA	NA	NA	NA	NA
NA	NA	NA	NA	NA	NA
NA	NA	NA	NA	NA	NA
NA	NA	NA	NA	NA	NA
NA	NA	NA	NA	NA	NA
NA	NA	NA	NA	NA	NA
NA	NA	NA	NA	NA	NA
NA	NA	NA	NA	NA	NA
NA	NA	NA	NA	NA	NA
0	0	NA	NA	NA	NA
0	0	NA	NA	NA	NA
NA	NA	NA	NA	NA	NA
NA	NA	NA	NA	NA	NA
NA	NA	NA	NA	NA	NA
NA	NA	NA	NA	NA	NA
100	8	NA	NA	NA	NA
100	8	NA	NA	NA	NA
NA	NA	NA	NA	NA	NA
NA	NA	NA	NA	NA	NA
NA	NA	NA	NA	NA	NA
NA	NA	NA	NA	NA	NA
NA	NA	NA	NA	NA	NA

NA	NA	NA	NA	NA	NA
NA	NA	NA	NA	NA	NA
NA	NA	NA	NA	NA	NA
NA	NA	NA	NA	NA	NA
NA	NA	NA	NA	NA	NA
NA	NA	NA	NA	NA	NA
NA	NA	NA	NA	NA	NA
NA	NA	NA	NA	NA	NA
NA	NA	NA	NA	NA	NA
NA	NA	NA	NA	NA	NA
NA	NA	NA	NA	NA	NA
NA	NA	NA	NA	NA	NA
NA	NA	NA	NA	NA	NA
100	7	NA	NA	NA	NA
NA	NA	NA	NA	NA	NA
NA	NA	NA	NA	NA	NA
NA	NA	NA	NA	NA	NA
0	0	NA	NA	NA	NA
NA	NA	NA	NA	NA	NA
0	0	NA	NA	NA	NA
0	0	NA	NA	NA	NA
100	4	NA	NA	NA	NA
0	0	NA	NA	NA	NA
0	0	NA	NA	NA	NA
0	0	NA	NA	NA	NA
0	0	NA	NA	NA	NA
0	0	NA	NA	NA	NA
0	0	NA	NA	NA	NA
20	4	NA	NA	NA	NA
NA	NA	NA	NA	NA	NA
NA	NA	NA	NA	NA	NA
NA	NA	NA	NA	NA	NA
NA	NA	NA	NA	NA	NA
NA	NA	NA	NA	NA	NA
NA	NA	NA	NA	NA	NA
NA	NA	NA	NA	NA	NA
NA	NA	NA	NA	NA	NA
NA	NA	NA	NA	NA	NA
NA	NA	NA	NA	NA	NA
NA	NA	NA	NA	NA	NA
NA	NA	NA	NA	NA	NA
NA	NA	NA	NA	NA	NA
NA	NA	NA	NA	NA	NA
NA	NA	NA	NA	NA	NA
NA	NA	NA	NA	NA	NA
NA	NA	NA	NA	NA	NA
0	0	NA	NA	NA	NA
NA	NA	NA	NA	NA	NA
NA	NA	NA	NA	NA	NA
NA	NA	NA	NA	NA	NA
NA	NA	NA	NA	NA	NA
NA	NA	NA	NA	NA	NA
NA	NA	NA	NA	NA	NA

0	0	NA	NA	NA	NA
0	0	NA	NA	NA	NA
NA	NA	NA	NA	NA	NA
NA	NA	NA	NA	NA	NA
NA	NA	NA	NA	NA	NA
0	0	NA	NA	NA	NA
NA	NA	NA	NA	NA	NA
NA	NA	NA	NA	NA	NA
NA	NA	NA	NA	NA	NA
NA	NA	NA	NA	NA	NA
NA	NA	NA	NA	NA	NA
NA	NA	NA	NA	NA	NA
NA	NA	NA	NA	NA	NA
NA	NA	NA	NA	NA	NA
NA	NA	NA	NA	NA	NA
NA	NA	NA	NA	NA	NA
100	4	NA	NA	NA	NA
0	0	NA	NA	NA	NA
NA	NA	NA	NA	NA	NA
NA	NA	NA	NA	NA	NA
0	0	NA	NA	NA	NA
0	0	NA	NA	NA	NA
NA	NA	NA	NA	NA	NA
NA	NA	NA	NA	NA	NA
0	0	NA	NA	NA	NA
0	0	NA	NA	NA	NA
0	0	NA	NA	NA	NA
NA	NA	NA	NA	NA	NA
40	4	NA	NA	NA	NA
20	3	NA	NA	NA	NA
100	2	NA	NA	NA	NA
100	2	NA	NA	NA	NA
100	1	NA	NA	NA	NA
100	2	NA	NA	NA	NA
100	3	NA	NA	NA	NA
100	2	NA	NA	NA	NA
10	2	NA	NA	NA	NA
NA	NA	NA	NA	NA	NA
NA	NA	NA	NA	NA	NA
NA	NA	NA	NA	NA	NA
NA	NA	NA	NA	NA	NA
NA	NA	NA	NA	NA	NA
100	3	NA	NA	NA	NA
NA	NA	NA	NA	NA	NA
NA	NA	NA	NA	NA	NA
NA	NA	NA	NA	NA	NA
100	7	NA	NA	NA	NA
50	4	NA	NA	NA	NA
0	0	NA	NA	NA	NA
0	0	NA	NA	NA	NA
0	0	NA	NA	NA	NA
0	0	NA	NA	NA	NA
0	0	NA	NA	NA	NA

80	3	NA	NA	NA	NA
80	3	NA	NA	NA	NA
0	0	NA	NA	NA	NA
0	0	NA	NA	NA	NA
0	0	NA	NA	NA	NA
0	0	NA	NA	NA	NA
0	0	NA	NA	NA	NA

% Cytoplasme	Cytoplasm intensity	Notes
NA	NA	
NA	NA	
NA	NA	
NA	NA	
NA	NA	
NA	NA	
NA	NA	
NA	NA	
NA	NA	
NA	NA	
NA	NA	
NA	NA	
NA	NA	
NA	NA	
NA	NA	
NA	NA	
NA	NA	
NA	NA	
NA	NA	
NA	NA	
NA	NA	
NA	NA	
NA	NA	
NA	NA	
NA	NA	
NA	NA	
NA	NA	
NA	NA	
NA	NA	sparse cytoplasmatic inclusion non specific staining
NA	NA	
NA	NA	
NA	NA	
NA	NA	
NA	NA	
NA	NA	
0	0	
NA	NA	
NA	NA	
NA	NA	
NA	NA	
NA	NA	
NA	NA	

NA	NA	
NA	NA	
NA	NA	
NA	NA	
NA	NA	
NA	NA	
NA	NA	
NA	NA	
NA	NA	
NA	NA	
NA	NA	
NA	NA	
NA	NA	
NA	NA	
NA	NA	
NA	NA	
NA	NA	
NA	NA	
NA	NA	
NA	NA	
NA	NA	
NA	NA	
NA	NA	
NA	NA	
NA	NA	
NA	NA	
NA	NA	
NA	NA	
NA	NA	
NA	NA	
NA	NA	
NA	NA	
NA	NA	no tissue core
NA	NA	
NA	NA	
NA	NA	
NA	NA	
NA	NA	
NA	NA	
NA	NA	
NA	NA	
NA	NA	
NA	NA	
NA	NA	
NA	NA	
NA	NA	
NA	NA	
NA	NA	
NA	NA	no tissue core
NA	NA	
NA	NA	
NA	NA	
NA	NA	

NA	NA	
NA	NA	
NA	NA	
NA	NA	no tissue core
NA	NA	matrix staining only
NA	NA	
NA	NA	
NA	NA	
NA	NA	
NA	NA	
NA	NA	
NA	NA	
NA	NA	
NA	NA	
NA	NA	
NA	NA	
NA	NA	
NA	NA	
NA	NA	
NA	NA	
NA	NA	
NA	NA	
NA	NA	
NA	NA	
NA	NA	
NA	NA	
NA	NA	
NA	NA	
NA	NA	
NA	NA	
NA	NA	
NA	NA	
NA	NA	
NA	NA	
NA	NA	
NA	NA	
NA	NA	Caultry Artifact, excluded
NA	NA	
NA	NA	
NA	NA	No tissue core, excluded
NA	NA	
NA	NA	
NA	NA	
NA	NA	
NA	NA	
NA	NA	
NA	NA	
NA	NA	
NA	NA	
NA	NA	
NA	NA	
NA	NA	
NA	NA	

NA	NA	
NA	NA	
NA	NA	
NA	NA	
NA	NA	
NA	NA	
NA	NA	
NA	NA	
NA	NA	
NA	NA	
NA	NA	
NA	NA	
NA	NA	
NA	NA	
NA	NA	
NA	NA	
NA	NA	
NA	NA	
NA	NA	
NA	NA	
NA	NA	
NA	NA	
NA	NA	
NA	NA	
NA	NA	
NA	NA	
NA	NA	
NA	NA	Torn tissue core, excluded
NA	NA	
NA	NA	100% fibrilar, int 2
NA	NA	100% fibrilar, int 2
NA	NA	100% fibrilar, int 2
NA	NA	100% fibrilar, int 2
NA	NA	100% fibrilar, int 2
NA	NA	100% fibrilar, int 2
NA	NA	100% fibrilar, int 2
NA	NA	100% fibrilar, int 1
NA	NA	100% fibrilar, int 1
NA	NA	100% fibrilar, int 1
NA	NA	100% fibrilar, int 2
NA	NA	100% fibrilar, int 3
NA	NA	100% fibrilar, int 3
NA	NA	100% fibrilar, int 2
NA	NA	100% fibrilar, int 1
NA	NA	100% fibrilar, int 2
NA	NA	100% fibrilar, int 2
NA	NA	fibrilar 0
NA	NA	fibrilar 0
NA	NA	100% fibrilar, int 1
NA	NA	100% fibrilar, int 1
NA	NA	fibrilar 0
NA	NA	fibrilar 0
NA	NA	100% fibrilar, int 2

NA	NA	100% fibrilar, int 2
NA	NA	100% fibrilar, int 2
NA	NA	100% fibrilar, int 2
NA	NA	100% fibrilar, int 2
NA	NA	100% fibrilar, int 2
NA	NA	100% fibrilar, int 2
NA	NA	100% fibrilar, int 2

Analysis 1	
Analyst	Date
Daria Kozlova MD	2022/3/14
Daria Kozlova MD	2022/3/14
Daria Kozlova MD	2022/3/14
Daria Kozlova MD	2022/3/14
Daria Kozlova MD	2022/3/14
Daria Kozlova MD	2022/3/14
Daria Kozlova MD	2022/3/14
Daria Kozlova MD	2022/3/14
Daria Kozlova MD	2022/3/14
Daria Kozlova MD	2022/3/14
Daria Kozlova MD	2022/3/14
Daria Kozlova MD	2022/3/14
Daria Kozlova MD	2022/3/14
Daria Kozlova MD	2022/3/14
Daria Kozlova MD	2022/3/14
Daria Kozlova MD	2022/3/14
Daria Kozlova MD	2022/3/14
Daria Kozlova MD	2022/3/14
Daria Kozlova MD	2022/3/14
Daria Kozlova MD	2022/3/14
Daria Kozlova MD	2022/3/14
Daria Kozlova MD	2022/3/14
Daria Kozlova MD	2022/3/14
Daria Kozlova MD	2022/3/14
Daria Kozlova MD	2022/3/14
Daria Kozlova MD	2022/3/14
Daria Kozlova MD	2022/3/14
Daria Kozlova MD	2022/3/14
Daria Kozlova MD	2022/3/14
Daria Kozlova MD	2022/3/14
Daria Kozlova MD	2022/3/14
Daria Kozlova MD	2022/3/14
Daria Kozlova MD	2022/3/14
Daria Kozlova MD	2022/3/14
Daria Kozlova MD	2022/3/14
Daria Kozlova MD	2022/3/14
Daria Kozlova MD	2022/3/14
Daria Kozlova MD	2022/3/14
Daria Kozlova MD	2022/3/14
Daria Kozlova MD	2022/3/14
Daria Kozlova MD	2022/3/14
Daria Kozlova MD	2022/3/14

Daria Kozlova MD	2022/3/14
Daria Kozlova MD	2022/3/14
Daria Kozlova MD	2022/3/14
Daria Kozlova MD	2022/3/14
Daria Kozlova MD	2022/3/14
Daria Kozlova MD	2022/3/14
Daria Kozlova MD	2022/3/14
Daria Kozlova MD	2022/3/14
Daria Kozlova MD	2022/3/14
Daria Kozlova MD	2022/3/14
Daria Kozlova MD	2022/3/14
Daria Kozlova MD	2022/3/14
Daria Kozlova MD	2022/3/14
Daria Kozlova MD	2022/3/14
Daria Kozlova MD	2022/3/14
Daria Kozlova MD	2022/3/14
Daria Kozlova MD	2022/3/14
Daria Kozlova MD	2022/3/14
Daria Kozlova MD	2022/3/14
Daria Kozlova MD	2022/3/14
Daria Kozlova MD	2022/3/14
Daria Kozlova MD	2022/3/14
Daria Kozlova MD	2022/3/14
Daria Kozlova MD	2022/3/14
Daria Kozlova MD	2022/3/14
Daria Kozlova MD	2022/3/14
Daria Kozlova MD	2022/3/14
Daria Kozlova MD	2022/3/14
Daria Kozlova MD	2022/3/14
Daria Kozlova MD	2022/3/14
Daria Kozlova MD	2022/3/14
Daria Kozlova MD	2022/3/14
Daria Kozlova MD	2022/3/14
Daria Kozlova MD	2022/3/14
Daria Kozlova MD	2022/3/14
Daria Kozlova MD	2022/3/14
Daria Kozlova MD	2022/3/14
Daria Kozlova MD	2022/3/14
Daria Kozlova MD	2022/3/14
Daria Kozlova MD	2022/3/14
Daria Kozlova MD	2022/3/14
Daria Kozlova MD	2022/3/14
Daria Kozlova MD	2022/3/14
Daria Kozlova MD	2022/3/14
Daria Kozlova MD	2022/3/14
Daria Kozlova MD	2022/3/14
Daria Kozlova MD	2022/3/14
Daria Kozlova MD	2022/3/14
Daria Kozlova MD	2022/3/14
Daria Kozlova MD	2022/3/14
Daria Kozlova MD	2022/3/14
Daria Kozlova MD	2022/3/14
Daria Kozlova MD	2022/3/14

Daria Kozlova MD	2022/3/14
Daria Kozlova MD	2022/3/14
Daria Kozlova MD	2022/3/14
Daria Kozlova MD	2022/3/14
Daria Kozlova MD	2022/3/14
Daria Kozlova MD	2022/3/14
Daria Kozlova MD	2022/3/14
Daria Kozlova MD	2022/3/14
Daria Kozlova MD	2022/3/14
Daria Kozlova MD	2022/3/14
Daria Kozlova MD	2022/3/14
Daria Kozlova MD	2022/3/14
Daria Kozlova MD	2022/3/14
Daria Kozlova MD	2022/3/14
Daria Kozlova MD	2022/3/14
Daria Kozlova MD	2022/3/14
Daria Kozlova MD	2022/3/14
Daria Kozlova MD	2022/3/14
Daria Kozlova MD	2022/3/20
Daria Kozlova MD	2022/3/20
Daria Kozlova MD	2022/3/20
Daria Kozlova MD	2022/3/20
Daria Kozlova MD	2022/3/20
Daria Kozlova MD	2022/3/20
Daria Kozlova MD	2022/3/20
Daria Kozlova MD	2022/3/20
Daria Kozlova MD	2022/3/20
Daria Kozlova MD	2022/3/20
Daria Kozlova MD	2022/3/20
Daria Kozlova MD	2022/3/20
Daria Kozlova MD	2022/3/20
Daria Kozlova MD	2022/3/20
Daria Kozlova MD	2022/3/20
Daria Kozlova MD	2022/3/20
Daria Kozlova MD	2022/3/20
Daria Kozlova MD	2022/3/20
Daria Kozlova MD	2022/3/20
Daria Kozlova MD	2022/3/20
Daria Kozlova MD	2022/3/20
Daria Kozlova MD	2022/3/20
Daria Kozlova MD	2022/3/20
Daria Kozlova MD	2022/3/20
Daria Kozlova MD	2022/3/20
Daria Kozlova MD	2022/3/20
Daria Kozlova MD	2022/3/20
Daria Kozlova MD	2022/3/20
Daria Kozlova MD	2022/3/20
Daria Kozlova MD	2022/3/20
Daria Kozlova MD	2022/3/20
Daria Kozlova MD	2022/3/20
Daria Kozlova MD	2022/3/20
Daria Kozlova MD	2022/3/20
Daria Kozlova MD	2022/3/20

Daria Kozlova MD	2022/3/20
Daria Kozlova MD	2022/3/20
Daria Kozlova MD	2022/3/20
Daria Kozlova MD	2022/3/20
Daria Kozlova MD	2022/3/20
Daria Kozlova MD	2022/3/20
Daria Kozlova MD	2022/3/20
Daria Kozlova MD	2022/3/20
Daria Kozlova MD	2022/3/20
Daria Kozlova MD	2022/3/20
Daria Kozlova MD	2022/3/20
Daria Kozlova MD	2022/3/20
Daria Kozlova MD	2022/3/20
Daria Kozlova MD	2022/3/20
Daria Kozlova MD	2022/3/20
Daria Kozlova MD	2022/3/20
Daria Kozlova MD	2022/3/20
Daria Kozlova MD	2022/3/20
Daria Kozlova MD	2022/3/20
Daria Kozlova MD	2022/3/20
Daria Kozlova MD	2022/3/20
Daria Kozlova MD	2022/3/20
Daria Kozlova MD	2022/3/20
Daria Kozlova MD	2022/3/20
Daria Kozlova MD	2022/3/20
Daria Kozlova MD	2022/3/20
Daria Kozlova MD	2022/3/20
Daria Kozlova MD	2022/3/20
Daria Kozlova MD	2022/3/20
Daria Kozlova MD	2022/3/20
Daria Kozlova MD	2022/3/20
Daria Kozlova MD	2022/3/20
Daria Kozlova MD	2022/3/20
Daria Kozlova MD	2022/3/20
Daria Kozlova MD	2022/3/20
Daria Kozlova MD	2022/3/20
Daria Kozlova MD	2022/3/20
Daria Kozlova MD	2022/3/20
Daria Kozlova MD	2022/3/20
Daria Kozlova MD	2022/3/20
Daria Kozlova MD	2022/3/20
Daria Kozlova MD	2022/3/20
Daria Kozlova MD	2022/3/20
Daria Kozlova MD	2022/3/20
Daria Kozlova MD	2022/3/20
Daria Kozlova MD	2022/3/20
Daria Kozlova MD	2022/3/20
Daria Kozlova MD	2022/3/20
Daria Kozlova MD	2022/3/20
Daria Kozlova MD	2022/3/20
Daria Kozlova MD	2022/3/20
Daria Kozlova MD	2022/3/20
Daria Kozlova MD	2022/3/20

Daria Kozlova MD	2022/3/20
Daria Kozlova MD	2022/3/20
Daria Kozlova MD	2022/3/20
Daria Kozlova MD	2022/3/20
Daria Kozlova MD	2022/3/20
Daria Kozlova MD	2022/3/20
Daria Kozlova MD	2022/3/20
